# Simultaneous confidence regions for image excursion sets: A validation study with applications in fMRI

**DOI:** 10.1162/IMAG.a.1044

**Published:** 2025-12-05

**Authors:** Jiyue Qin, Samuel Davenport, Armin Schwartzman

**Affiliations:** Division of Biostatistics and Bioinformatics, Herbert Wertheim School of Public Health and Human Longevity Science, University of California, San Diego, CA, United States; Halıcıoğlu Data Science Institute, University of California, San Diego, CA, United States

**Keywords:** simultaneous confidence regions, bootstrap, simultaneous confidence band, fMRI

## Abstract

Functional Magnetic Resonance Imaging (fMRI) is commonly used to localize brain regions activated during a task. Methods have been developed for constructing confidence regions of image excursion sets, allowing inference on brain regions exceeding non-zero activation thresholds. However, these methods have been limited to a single predefined threshold and brain volume data, overlooking more sensitive cortical surface analyses. We present an approach that constructs simultaneous confidence regions (SCRs) which are valid for all possible activation thresholds and are applicable to both volume and surface data. This approach is based on a recent method that constructs SCRs from simultaneous confidence bands (SCBs), obtained by using the bootstrap on 1D and 2D images. To extend this method to fMRI studies, we evaluate the validity of the bootstrap with fMRI data through extensive 2D simulations. Six bootstrap variants, including the nonparametric bootstrap and multiplier bootstrap, are compared. The Rademacher multiplier bootstrap-t performs the best, achieving a coverage rate close to the nominal level with sample sizes as low as 10. We further validate our approach using realistic noise simulations obtained by resampling resting-state 3D fMRI data, a technique that has become the gold standard in the field. Moreover, our implementation handles data of any dimension and is equipped with interactive visualization tools designed for fMRI analysis. We apply our approach to task fMRI volume data and surface data from the Human Connectome Project, showcasing the method’s utility.

## 1 Introduction

Functional Magnetic Resonance Imaging (fMRI) is a widely used noninvasive neuroimaging technique for measuring brain activity by detecting changes in blood flow (Lindquist, 2008). During an fMRI experiment, a sequence of brain scans is captured while a participant performs a task. Each individual scan corresponds to a 3D image of the brain comprising more than 200,000 voxels (Cremers et al., 2017), where the image intensity at each location is driven by blood-oxygen level-dependent (BOLD) signal, which is a proxy for neural activity. A first-level analysis is performed to create a 3D contrast image, which represents the change in brain activity at each voxel, in units of percentage BOLD change (Lindquist, 2008). Traditionally, task-activated brain regions are identified using voxel-wise or cluster-wise inference (Nichols, 2012). Voxel-wise inference involves conducting hypothesis tests on the %BOLD change for each voxel separately adjusting for multiple testing. In contrast, cluster-wise inference declares a cluster significant if its size (i.e., number of voxels) exceeds some threshold.

While standard, the testing approach has several limitations. First, it is typically conducted under the null hypothesis that the change in brain activity is zero. However, in practice, a large amount of the brain may exhibit non-zero albeit low activation which may or may not be of interest (Gross & Binder, 2014). This means that increasing the sample size will result in rejecting the null in increasingly more locations, losing spatial precision (Bowring et al., 2019; Davenport et al., 2022). Instead, researchers may seek to identify brain regions where the activation is particularly strong, for example, greater than 2% BOLD change. Second, with hypothesis testing, fMRI results are typically presented with thresholded color-coded statistical maps that only highlight significant regions (Poldrack et al., 2008). However, test statistics are unitless and do not provide a clinical interpretation, prompting recommendations on more emphasis on effect estimates (Chen et al., 2017; Taylor et al., 2023). Moreover, highlighting only significant areas overlooks areas that have large changes but are statistically insignificant due to insufficient power (Greenland et al., 2016).

Furthermore, in cluster-wise inference, a significant cluster-level p value only indicates activation occurred for at least one voxel in that cluster without identifying what voxels are activated. This leads to the spatial specificity paradox, where a larger cluster conveys less information about the location of activation (Woo et al., 2014). To address this issue, methods providing simultaneous lower bounds for true discovery proportion (TDP) have been developed in neuroimaging data (Andreella et al., 2023; Blain et al., 2022; Davenport et al., 2025; Goeman et al., 2023; Rosenblatt et al., 2018), mostly based on theory from multiple testing (Blanchard et al., 2020; Goeman & Solari, 2011; Katsevich & Ramdas, 2020). However, these methods do not provide information about the spatial variation of significant clusters, that is, if an fMRI study were repeated multiple times, the size and shape of significant clusters would likely vary, but existing methods do not reflect this variation (Bowring et al., 2019). Additionally, some methods may suffer from low statistical power (e.g., Rosenblatt et al., 2018), or are computationally intensive (e.g., the more accurate heuristic algorithm in Goeman et al., 2023).

**Fig. 1.**
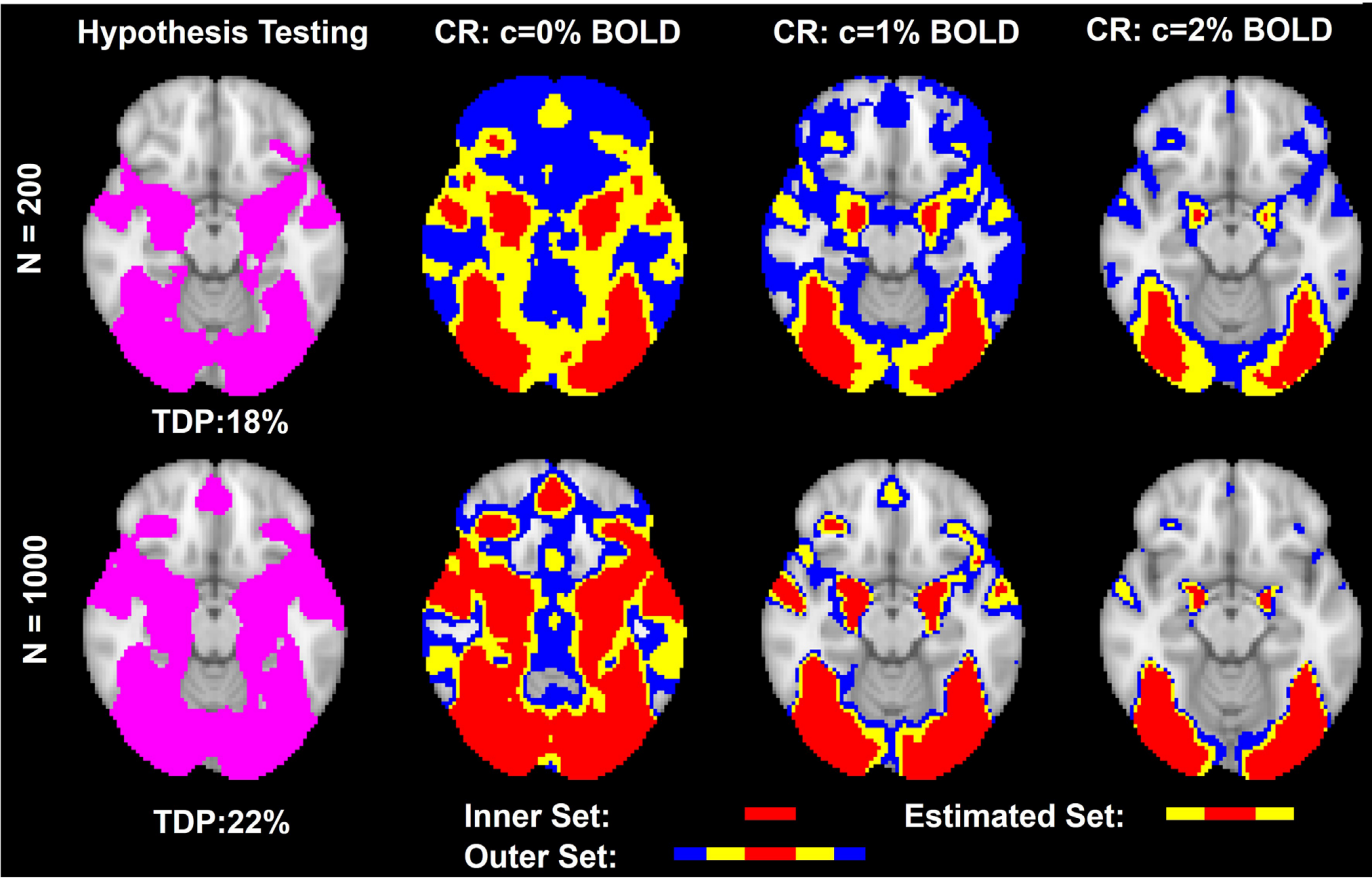
Activated brain regions obtained using hypothesis testing and the confidence regions (CR) approach with thresholds of 0, 1, and 2, with sample sizes of 200 and 1,000. The data are from the Hariri faces/shapes “emotion” task in UK Biobank. Hypothesis testing was conducted using permutation-based cluster-wise inference at a cluster defining threshold of 3.1. Lower bounds for the True Discovery Proportion (TDP) were calculated using the method from Goeman et al. (2023). For the CR results, the red region, union of red and yellow region, union of red and yellow and blue region represent the inner set, estimated set, outer set, respectively. To interpret the CR results, for example, at c=2% BOLD change, we can state with at least 95% confidence that the true brain regions with more than 2% BOLD change lie between the inner set and the outer set. When the sample size is large, hypothesis testing finds a large significant cluster with a small TDP, losing spatial precision. In contrast, CRs using a non-zero threshold yield more informative and interpretable results.

Instead, the problem of activation localization is more naturally formulated as finding confidence regions for the true activated region exceeding a threshold. This approach, analogous to presenting a confidence interval, allows non-zero thresholds, preserves information on the effect estimate, and facilitates interpretation. Figure 1 illustrates a comparison between the traditional hypothesis testing approach and the confidence regions approach.

Sommerfeld et al. (2018) proposed a spatial inference method for constructing confidence regions, which provide spatial uncertainty in the estimation of excursion sets of the mean function in images. This method was later refined and applied to fMRI data by Bowring et al. (2019), allowing inference on brain regions with non-zero activation thresholds. However, this general approach is limited to one predetermined activation threshold. In practice, deciding on a reasonable threshold beforehand may be difficult, and researchers are inclined to explore various thresholds, which necessitates addressing the issue of multiple testing over thresholds (Bowring et al., 2019). Moreover, this approach can only be applied to volume and not cortical surface data. This is a critical limitation since surface-based analyses, recognized for their greater sensitivity, reliability, and specificity than volume-based methods, have received increasing attention (Brodoehl et al., 2020; Tucholka et al., 2012). Bayesian approaches which provide posterior confidence regions for excursion sets of cortical surface data have been proposed (Mejia et al., 2020; Spencer et al., 2022). However, these also consider a single threshold and rely on assumptions of stationarity and Gaussianity.

Recently Ren et al. (2024) proposed a method for constructing confidence regions (CRs) that remain valid for all possible thresholds, hence the name, “simultaneous confidence regions (SCRs).” In this method, CRs for any given threshold are obtained by inverting simultaneous confidence bands (SCBs) at that threshold. This process can be repeated for different thresholds, ensuring that all resulting CRs are valid simultaneously. The key step of this method is therefore the construction of valid SCBs, typically obtained via bootstrap techniques (Chang et al., 2017; Chernozhukov et al., 2013; Degras, 2011).

To extend the SCR method to fMRI studies, we need to ensure the validity of the bootstrap with fMRI data. Prior evaluations of the bootstrap have mostly used 1D or Gaussian models in simulations (Bowring et al., 2019; Telschow & Schwartzman, 2022), which fail to reflect the higher-dimensional, non-stationary, non-Gaussian nature of fMRI data (Davenport et al., 2023; Hanson & Bly, 2001; Wager et al., 2005). Eklund et al. (2016) emphasized that simulations under restrictive assumptions such as stationarity are insufficient to establish the validity of statistical methods in fMRI studies. They proposed using resting-state validations, which fit a fake task design to resting-state data in order to generate realistic noise and have become the gold standard for method validation in fMRI (Andreella et al., 2023; Davenport et al., 2023; Lohmann et al., 2018).

The contributions of this paper are as follows. First, we evaluate six bootstrap variants for constructing SCBs, including the nonparametric bootstrap and multiplier bootstrap, through extensive 2D simulations with Gaussian and non-Gaussian data. Compared to Bowring et al. (2019), our method uses the bootstrap to approximate the distribution of the maximum over the entire random field, while their approach targets the distribution of the maximum on the boundary of the excursion set and is valid only for a single pre-specified activation threshold. Additionally, our simulations evaluate more bootstrap variants and include non-Gaussian scenarios. We find that the Rademacher multiplier bootstrap-t performs the best, achieving a coverage rate close to the nominal level with sample sizes as low as 10. Second, we validate the corresponding coverage of the SCRs using realistic 3D resting-state fMRI data. Third, we have developed software that constructs confidence regions for data of any dimension, such as brain volume and surface data. Our software is equipped with visualization tools tailored for fMRI, including interactive apps that allow users to visualize activated brain regions as they adjust the activation threshold. Finally, we illustrate our approach with an application to both fMRI volume data and surface data from the Human Connectome Project.

We have implemented this method in the Python package SimuInf (Qin, 2024). A Matlab implementation is also available in the StatBrainz package (Davenport, 2024). Demonstrations of the interactive apps for volume and surface data analyses are provided in Figures 9 and 10. All the simulations and analyses were run on an Intel Core CPU@2.1 GHz with 16GB RAM.

## 2 Theory

### 2.1 Confidence regions for an excursion set

We follow the notations and theory in Ren et al. (2024). Let $S\subset {\mathbb{R}}^{D}$, D∈ℕ, be a domain and μ:S→ℝ be a signal of interest. In the context of fMRI, $S\subset {\mathbb{R}}^{3}$ corresponds to the set of voxels or vertices making up the brain and μ(s) represents the %BOLD change at voxel/vertex s∈S . The inverse image of μ under a set U⊂ℝ is defined as $\mu^{-1}(U)=\{s\in S:\mu (s)\in U\}$ . For a real number c, if U=[c,∞) , then $\mu^{-1}(U)$ is called the excursion set of μ above the level c. In fMRI, researchers aim to identify areas of the brain activated during a task. For instance, setting c=2 , the excursion set $\mu^{-1}[2,\infty )$ is the quantity of interest and represents brain areas with at least 2% BOLD change.

CRs quantify the uncertainty in estimating $\mu^{-1}[c,\infty )$ . They consist of an inner set, denoted as ${\hat{CR}}_{in}[c,\infty )$ , and an outer set, denoted as ${\hat{CR}}_{out}[c,\infty )$ , such that

$$\underset{N\to \infty }{\text{lim}}\mathbb{P}\left[{\hat{CR}}_{in}[c,\infty )\subseteq \mu^{-1}[c,\infty )\subseteq {\hat{CR}}_{out}[c,\infty )\right]=1-\alpha ,$$ (*)

where 1−α is the desired coverage level and α is typically set at 0.05. Of note, the inner and outer sets are estimated from data, making them random quantities depending on sample size N. Throughout the paper, we use^(as in ${\hat{CR}}_{in}$ ) to denote quantities estimated from a sample of size N. As indicated in Equation (*), we focus on asymptotic CRs which achieve target coverage level when sample size is large. While Bowring et al. (2019) used the terms “upper” and “lower” sets, we prefer the terms “inner” and “outer” to indicate that the inner set is contained within the outer set. Ren et al. (2024) used the term “confidence sets” to present the theory in a general and abstract setting; however, we favor the term “confidence regions” as it emphasizes that they quantify spatial uncertainty and is more consistent with the terminology used in the fMRI research community, where it is common to use spatial terms such as “regions of activation” and “regions of interest.” Furthermore, our terminology is consistent with Sommerfeld et al. (2018), who first introduced this type of spatial inference.

### 2.2 Constructing simultaneous confidence regions by inverting the SCB

To obtain SCRs suitable for application in brain imaging, we follow the approach of Ren et al. (2024). They proposed constructing CRs of $\mu^{-1}[c,\infty )$ that are simultaneously valid for all c∈ℝ by inverting an SCB of μ(s) . An asymptotic SCB consists of a lower function ${\hat{B}}_{l}(s)$ and an upper function ${\hat{B}}_{u}(s)$ such that:

$$\underset{N\to \infty }{\text{lim}}\mathbb{P}\left[\text{for all }s\in S,{\hat{B}}_{l}(s)\leq \mu (s)\leq {\hat{B}}_{u}(s)\right]=1-\alpha .$$

Given an asymptotic SCB, CRs can be calculated as ${\hat{B}}_{l}^{-1}[c,\infty )$ for the inner set and ${\hat{B}}_{u}^{-1}[c,\infty )$ for the outer set. Theorem 1 in Ren et al. (2024) established an equivalence between the SCB and the CRs, that is:

$$\begin{array}{l} \mathbb{P}[\text{for all }c\in \mathbb{R},{\hat{B}}_{l}^{-1}[c,\infty )\subseteq \mu^{-1}[c,\infty )\subseteq {\hat{B}}_{u}^{-1}[c,\infty )]\\ \;\;\;=\mathbb{P}[\text{for all }s\in S,{\hat{B}}_{l}(s)\leq \mu (s)\leq {\hat{B}}_{u}(s)]. \end{array}$$

Of note, as can be seen from their proof of Theorem 1, in fact, the corresponding two events are equivalent, that is:

$$\begin{array}{l} \left\{\text{for all }c\in \mathbb{R},{\hat{B}}_{l}^{-1}[c,\infty )\subseteq \mu^{-1}[c,\infty )\subseteq {\hat{B}}_{u}^{-1}[c,\infty )]\right\}\\ \;\;\;=\{\text{for all }s\in S,{\hat{B}}_{l}(s)\leq \mu (s)\leq {\hat{B}}_{u}(s)\}. \end{array}$$

These CRs are valid for all c∈ℝ , hence the name, “simultaneous confidence regions.” That is, we have:

$$\underset{N\to \infty }{\text{lim}}\mathbb{P}\left[\text{for all }c\in \mathbb{R},{\hat{B}}_{l}^{-1}[c,\infty )\subseteq \mu^{-1}[c,\infty )\subseteq {\hat{B}}_{u}^{-1}[c,\infty )\right]=1-\alpha .$$

**Fig. 2.**
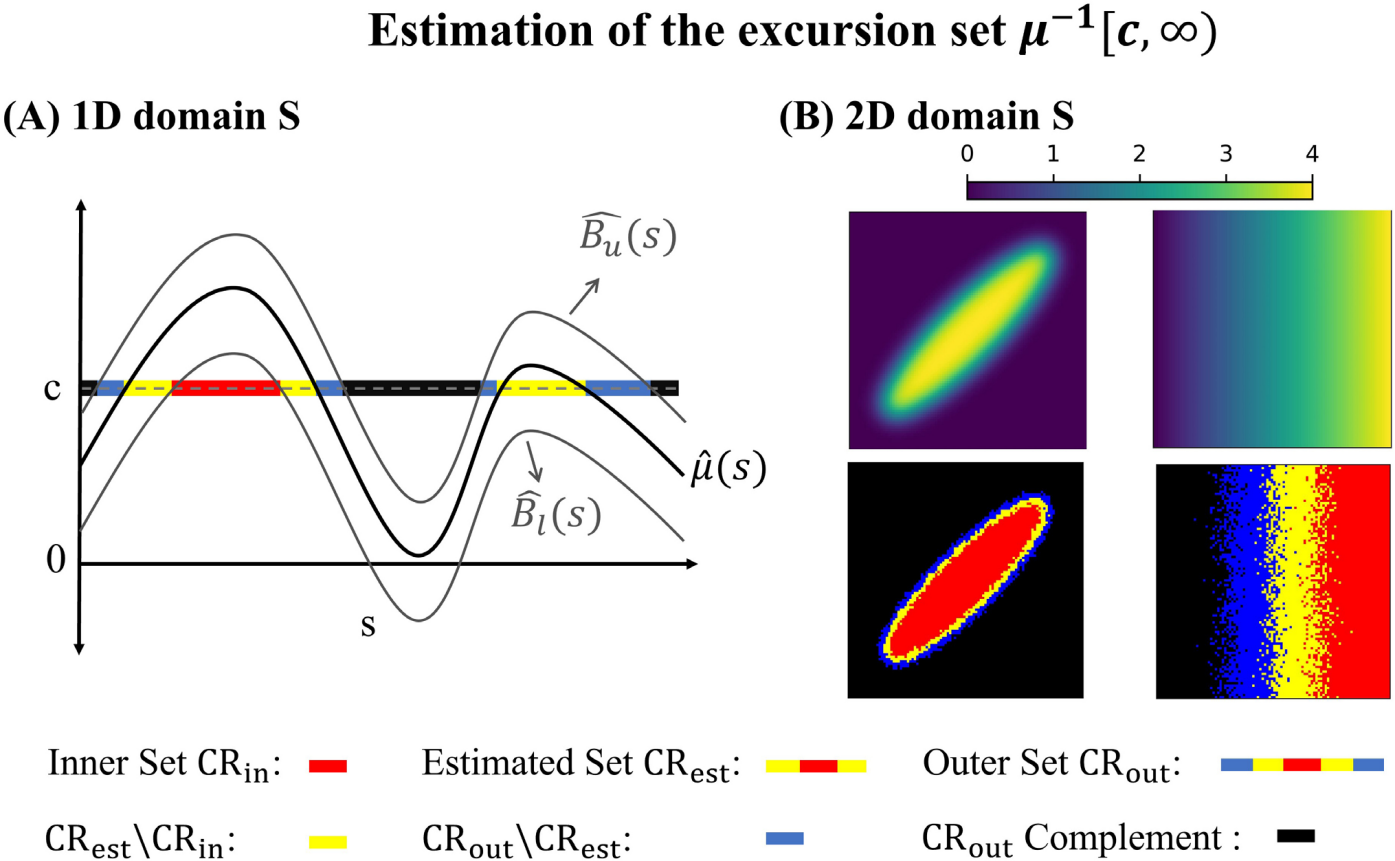
Illustration of the simultaneous confidence region (SCR) method with a 1D function (A) and a 2D function (B). The red region, union of red and yellow region, union of red, yellow and blue region represent the inner set ${\text{CR}}_{\text{in}}$ , estimated set ${\text{CR}}_{\text{est}}$ , and outer set ${\text{CR}}_{\text{out}}$ , respectively. Correspondingly, the yellow, blue, and black region represent ${\text{CR}}_{\text{est}}{\text{\textbackslash{}CR}}_{\text{in}}$ (i.e., elements in ${\text{CR}}_{\text{est}}$ but not in ${\text{CR}}_{\text{in}}$ ), ${\text{CR}}_{\text{out}}{\text{\textbackslash{}CR}}_{\text{est}}$ (i.e., elements in ${\text{CR}}_{\text{out}}$ but not in ${\text{CR}}_{\text{est}}$ ) and ${\text{CR}}_{\text{out}}$ complement, respectively. In (A), the black curve represents the estimator of μ(s) . The two gray curves represent the simultaneous confidence band (SCB) of μ(s) . In (B), the top two panels show two examples for μ(s) , taking the shape of an ellipse and a ramp. The bottom two panels show their corresponding estimated excursion sets at c=2 and SCRs based on 40 samples from Model 1.

Figure 2A illustrates the idea of this method with a 1D function μ(s):s∈S⊂ℝ . To estimate the excursion set $\mu^{-1}[c,\infty )$ , we first calculate $\hat{\mu }(s)$ , the estimator of μ(s) . The SCB of μ(s) is then constructed, consisting of ${\hat{B}}_{l}(s)$ and ${\hat{B}}_{u}(s)$ . Finally, the inner, estimated, and outer sets are obtained by inverting $\hat{\mu }(s),{\hat{B}}_{l}(s),{\hat{B}}_{u}(s)$ respectively at the threshold c. With a 2D function, as depicted in Figure 2B, the estimated set and its SCRs can be obtained similarly.

### 2.3 SCB in functional signal-plus-noise models

We focus on signal-plus-noise models, which include regression models that are widely used in second-level fMRI data analyses (Mumford & Nichols, 2009). Let $Y_{1},\ldots ,Y_{N}\overset{i.i.d.}{∼}Y$ be an independent and identically distributed (i.i.d.) sample of random functions, where Y follows the following functional signal-plus-noise model:

$$Y(s)=\mu (s)+\sigma (s)Z(s),\;\text{for }s\in S\subset {\mathbb{R}}^{D}.$$ (1)

Here, μ(s) and σ(s) are fixed functions, Z(s) is a random function with mean zero and variance one for all s, ϵ(s)=σ(s)Z(s) is the noise function. Of note, we do not assume stationarity with respect to the argument s, a particular correlation structure, or a particular distribution (for example, Gaussian) on the noise field ϵ(s) .

Define the sample mean and sample variance as:

$${\hat{\mu }}_{N}(s)=\frac{1}{N}\sum_{n=1}^{N}Y_{n}(s),{\hat{\sigma }}_{N}^{2}(s)=\frac{1}{N-1}\sum_{n=1}^{N}{\left[Y_{n}(s)-{\hat{\mu }}_{N}(s)\right]}^{2}.$$

Of note, the subscript N in ${\hat{\mu }}_{N}(s),{\hat{\sigma }}_{N}^{2}(s)$ emphasizes that these estimators depend on the sample size N. If we can find $q_{\alpha }$ such that

$$\underset{N\to \infty }{\text{lim}}\mathbb{P}\left(\underset{s}{\text{sup}}\left|\frac{{\hat{\mu }}_{N}(s)-\mu (s)}{{\hat{\sigma }}_{N}(s)\;/\;\sqrt{N}}\right|\leq q_{\alpha }\right)=1-\alpha$$

then we have:

$$\underset{N\to \infty }{\text{lim}}\mathbb{P}\left(\text{for all }s\in S,{\hat{\mu }}_{N}\left(s\right)-q_{\alpha }\frac{{\hat{\sigma }}_{N}(s)}{\sqrt{N}}\leq \mu \left(s\right)\leq {\hat{\mu }}_{N}(s)+q_{\alpha }\frac{{\hat{\sigma }}_{N}(s)}{\sqrt{N}}\right)=1-\alpha$$

Therefore, an asymptotically valid Wald-based SCB of μ(s) is:

$$\text{SCB}(s)={\hat{\mu }}_{N}(s)\pm q_{\alpha }\frac{{\hat{\sigma }}_{N}(s)}{\sqrt{N}}.$$

Note $\frac{{\hat{\mu }}_{N}(s)-\mu (s)}{{\hat{\sigma }}_{N}(s)\;/\;\sqrt{N}}$ converges to a Gaussian random field. Thus, $q_{\alpha }$ represents the upper αth quantile of the supremum of the limiting Gaussian field, which is usually unknown and can be estimated from bootstrap methods as described in Section 2.4. We denote this estimator by ${\hat{q}}_{\alpha ,N}$ .

### 2.4 Variants of bootstrap methods

SCBs are typically constructed using bootstrap methods. We examine six bootstrap variants. In this section, we describe how two of the most widely used bootstrap methods can be used to provide the quantile ${\hat{q}}_{\alpha ,N}$ . These methods are adaptations of the nonparametric bootstrap (Efron, 1979) and wild bootstrap (Wu, 1986) for functional data. We conclude the section by summarizing additional variations on these bootstrap methods.

**Nonparametric bootstrap (Degras, 2011):**

1. Resample from $Y_{1},\ldots ,Y_{N}$ with replacement to produce a bootstrap sample $Y_{1}*,\ldots ,Y_{N}*$ .
2. Compute ${\hat{\mu }}_{N}^{*}(s)$ and ${\hat{\sigma }}_{N}^{*}(s)$ using the sample $Y_{1}*,\ldots ,Y_{N}*$ .
3. Compute $T*=\underset{s\in S}{\text{max}}\sqrt{N}\left|\frac{{\hat{\mu }}_{N}^{*}(s)-{\hat{\mu }}_{N}(s)}{{\hat{\sigma }}_{N}^{*}(s)}\right|$.
4. Repeat steps 1 to 3 many times to get the distribution of T* and set ${\hat{q}}_{\alpha ,N}$ to be the (1−α)th quantile of this distribution.

**Multiplier (or Wild) Bootstrap (Chang et al., 2017):**

1. Define residuals $R_{N}^{n}(s)=Y_{n}(s)-{\hat{\mu }}_{N}(s)$ , compute $R_{N}^{1},\ldots ,R_{N}^{N}$ and multipliers $g_{1},\ldots ,g_{N}\overset{i.i.d.}{∼}g$ with E[g]=0 and var[g]=1 to produce a bootstrap sample $g_{1}R_{N}^{1}(s),\ldots ,g_{N}R_{N}^{N}(s)$ . We consider two common choices of g: a standard Gaussian random variable or a Rademacher random variable, which takes values of 1 and -1 with probability 1 / 2 .
2. Compute ${\hat{\mu }}_{N}*(s)$ and ${\hat{\sigma }}_{N}*(s)$ from $g_{1}R_{N}^{1}(s),\ldots ,g_{N}R_{N}^{N}(s)$ .
3. Compute $T*=\underset{s\in S}{\text{max}}\sqrt{N}\left|\frac{{\hat{\mu }}_{N}^{*}(s)}{{\hat{\sigma }}_{N}^{*}(s)}\right|$.
4. Repeat steps 1 to 3 many times to get the distribution of T​* and set ${\hat{q}}_{\alpha ,N}$ to be the (1−α)th quantile of this distribution.

Of note, in both methods described above, the third step standardizes the bootstrap sample mean with bootstrap sample standard deviation (SD), akin to the calculation of a T score. An alternative approach is to standardize with the original sample SD, mirroring the calculation of a Z score (Chernozhukov et al., 2013; Sommerfeld et al., 2018). These two types of standardizations are referred to as T and Z standardization. We evaluate six bootstrap methods, which are a combination of three bootstrap types (nonparametric, Gaussian multiplier, Rademacher multiplier) and two standardization types (T, Z).

## 3 Methods

### 3.1 2D simulations

We conducted a series of 2D simulations to evaluate various bootstrap methods for constructing SCBs, assessing the following aspects: coverage rate, runtime, precision, and stability. We considered various scenarios and bootstrap methods, as detailed below. For all scenarios considered, the number of simulation replications was 1,000, the number of bootstrap samples was 1,000, and the significance level α was 0.05, corresponding to a target coverage level of 1−α=0.95 . Coverage rate was calculated as the proportion of simulation instances in which the true means at all grid points fell within their respective confidence bands, thereby assessing the simultaneous coverage across all grid points. Average runtime across the 1,000 simulation replications was calculated. Precision was assessed by the mean of the quantile ${\hat{q}}_{\alpha ,N}$ across the 1,000 simulation replications, where a smaller value corresponds to a narrower and thus more precise SCB. Stability was assessed by the standard deviation (SD) of ${\hat{q}}_{\alpha ,N}$ across the 1,000 replications, where a smaller value represents a more stable SCB.

In each simulation instance, the data were generated as an i.i.d. sample from Model 1. The following parameters were varied, leading to a combination of 640 scenarios:

- shape of the signal μ(s)∈ {ellipse, ramp}, as depicted in Figure 2B
- noise distribution before smoothing ∈ {Standard Gaussian, Student’s t with 3 degrees of freedom ($t_{3}$)}
- In detail, before smoothing, the ϵ(s) was generated as i.i.d. over s from the given distribution. The $t_{3}$ distribution was chosen since it approximates the noise distribution of fMRI data (Davenport et al., 2023)
- full width at half maximum (FWHM) in Gaussian kernel smoothing of the noise ∈ {0, 1, 2, 3, 4}
- Of note, smoothing introduces correlation in the noise ϵ(s) over s
- SD of the noise after smoothing ∈{1,10}
- Specifically, after smoothing, the noise ϵ(s) was normalized to have the same SD of 1 or 10 over s
- 2D image size ∈{50×50, 100×100}
- sample size ∈{10, 20, 40, 60, 80, 100, 150, 200}

For each scenario, we evaluated six bootstrap methods: three bootstrap types (nonparametric, Gaussian multiplier, Rademacher multiplier) × two standardization types (T, Z).

Since SCRs remain valid across all thresholds, when users have a pre-specified threshold, the CR method in Bowring et al. (2019) is expected to offer better spatial precision. To evaluate the trade-off between spatial precision and the greater flexibility of SCRs, we compare the SCR and CR methods at a pre-specified threshold through simulations. Specifically, since the inner set contains pixels detected with high confidence, we measure spatial precision using true positive detection rate, defined as the proportion of truly positive pixels in the inner set out of all pixels in the inner set, averaged across 1,000 simulations.

### 3.2 3D validations

In order to test the performance of the SCRs in realistic noise settings, we conducted resting state validations to assess the coverage rate of the SCBs and the resulting confidence regions. To do so we used 3D contrast images obtained from resting-state fMRI data of 198 healthy controls (Beijing dataset) from the 1,000 Functional Connectomes Project (Biswal et al., 2010). These images were processed using FSL (Jenkinson et al., 2012) by Eklund et al. (2016) using a fake task design consisting of a 10-second on/off block activity paradigm and a 4 mm FWHM smoothing. Since resting-state data should not contain systematic changes in brain activity, these contrast images are expected to have a mean of zero. A realistic signal was introduced by adding the average %BOLD change during the Hariri faces/shapes “emotion” task, from 4,000 UK Biobank participants (Alfaro-Almagro et al., 2018), to each 3D contrast image.

To evaluate the coverage rate for a sample of size N, in each analysis instance, N images were sampled without replacement from the 198 3D contrast images. SCBs were subsequently constructed using the Rademacher multiplier bootstrap-t and the confidence regions for various numbers of predefined thresholds were obtained. The Rademacher multiplier bootstrap-t was used since it achieved a coverage rate close to the nominal level in previous 2D simulations. This procedure was replicated 1,000 times, mimicking the regular Monte Carlo simulation but with realistic datasets. The coverage rate of the SCBs was calculated as described in Section 3.1.

The theory asserts that the coverage rate of the SCB equals that of the SCRs across all thresholds. Therefore, in practice, with any finite number of thresholds, the coverage rate of the SCRs is guaranteed to exceed the nominal level and approaches it as the number of thresholds increases. To illustrate this, we evaluated the coverage of the SCRs with increasing numbers of thresholds. The coverage rate of the SCRs was calculated as the proportion of analysis instances in which the true excursion set contained the inner set and was contained by the outer set for all predefined thresholds, thereby assessing the simultaneous coverage across thresholds. That is,

$$\begin{array}{l} \text{SCR coverage rate}=\#\{\text{Analysis Instance}:\;\\ \;\;\;\;\;\text{for all }c\in K,\;{\text{CR}}_{\text{in}}\subseteq \mu^{-1}[c,\infty )\subseteq {\text{CR}}_{\text{out}}\}\;/\;1000, \end{array}$$

where K is the set of predefined thresholds.

The thresholds considered were taken to be equidistant from -20 to 20, covering the range where the majority of the signal lies. Different sample sizes (10, 20, 30, 40, 50 ) and numbers of thresholds (5, 10, 50, 100, 1000 ) were examined to evaluate the method’s performance under different scenarios. Since the assumed activity paradigm in the first-level analysis may influence the results (Eklund et al., 2016), the above evaluations were repeated with contrast images generated with an event activity paradigm (1- to 4-second activation, 3- to 6-second rest, randomized), 4 mm FWHM using FSL.

### 3.3 Application to task fMRI volume and surface data

To illustrate the performance of the SCRs in practice, we applied them to volume and cortical surface task fMRI data from the Human Connectome Project (HCP). The sample included 78 unrelated subjects engaged in a working memory task. A second-level analysis was conducted on the 78 3D contrast images to determine the task-activated brain regions across the participants. A similar analysis was conducted for the 78 cortical surface images to determine activated surface areas. Detailed descriptions of the study protocol, task paradigm, and first-level analyses are available in Barch et al. (2013) and Glasser et al. (2013), with a brief summary provided below.

The task contained two runs, each consisting of four blocks. In each block, the participant undertook either a 2-back memory task or a 0-back control task. The experimental design was arranged such that, in each run, two blocks were designated to the 2-back memory task, and two blocks were designated to the 0-back control task. In each block, a participant was shown a stimuli image (a picture of a face or a place, for instance) and then asked to recall the image they were shown. They were either asked to recall the most recent image (the 0-back image) or the image shown to them two images prior (the 2-back image). First-level analyses were conducted independently for each participant using FSL, where the task design was regressed onto the BOLD response, generating a contrast image for each participant. These images represent the difference in BOLD response between the 2-back task and the 0-back task.

## 4 Results

### 4.1 2D simulations

**Fig. 3.**
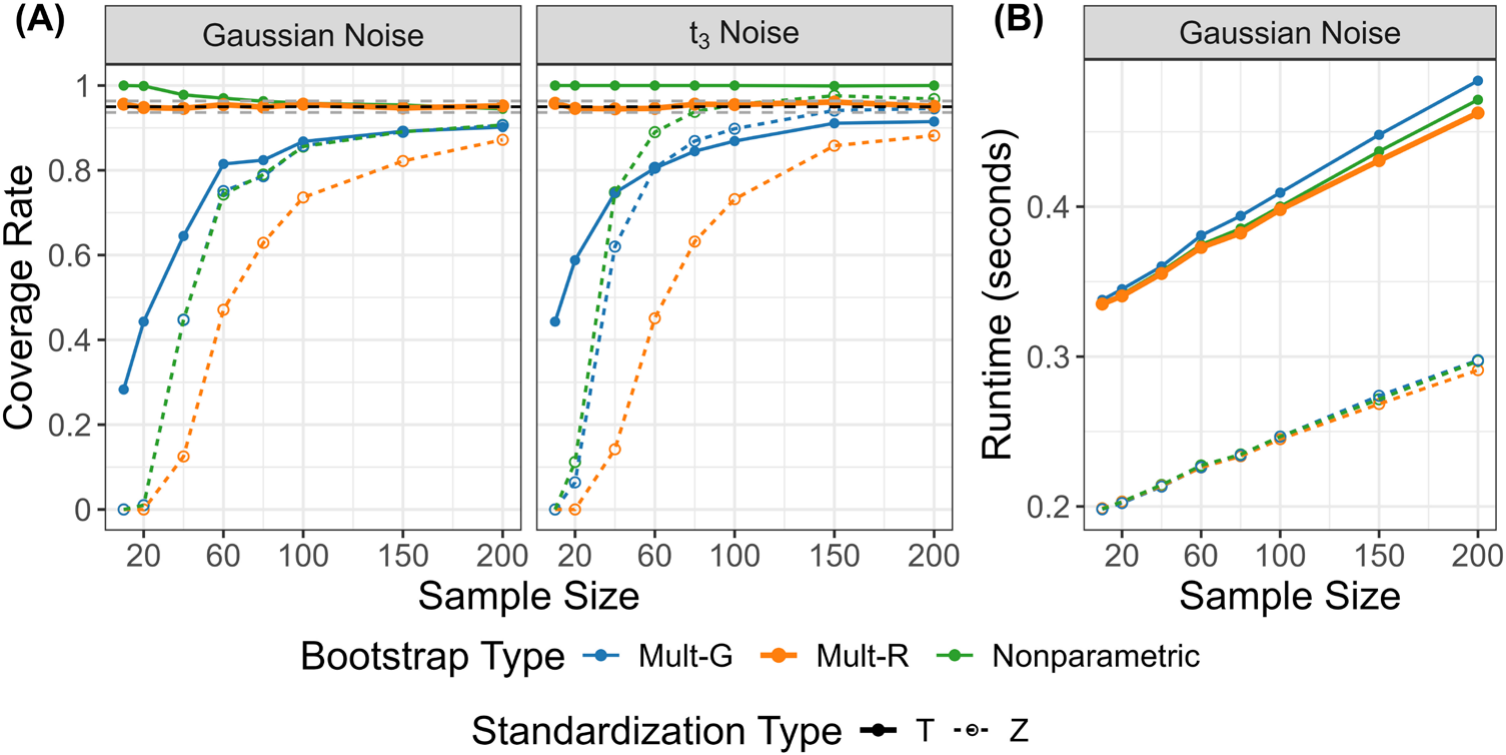
Results of 2D simulations on coverage rate (A) and runtime (B) under variations in sample size and noise distribution. Six bootstrap methods (3 bootstrap types × 2 standardization types) were evaluated. (A) The black dashed line indicates the target coverage rate of 0.95. The two gray dashed lines capture the uncertainty due to simulation and correspond to $0.95\pm 1.96\times \sqrt{0.95(1-0.95)\;/\;1000}$ . Of note, under the Gaussian noise, the coverage of Gaussian multiplier-z (blue dashed curve) is very similar to that of nonparametric-z (green dashed curve). Among these methods, the Rademacher multiplier bootstrap-t performs the best, achieving a coverage rate close to the target level under all variations considered. (B) Methods with Z standardization are faster than T standardization, independent of bootstrap type. Runtime results under $t_{3}$ noise are very similar and are thus omitted. Confidence intervals of runtime are not plotted since they are extremely narrow, due to the runtime being highly consistent across the 1,000 simulations. For example, the runtime at sample size of 100 with Rademacher multiplier-t was 0.398 seconds [95% CI: 0.397, 0.399].

The simulation results are presented for the scenarios with an ellipse shape, FWHM smoothing of 2, noise SD of 10, and an image size of 100×100 . Results in other scenarios are similar and are provided in the Supplementary File (Sections S2.1 to S2.4). In assessing the coverage rate, as depicted in Figure 3A, among all the methods evaluated, the Rademacher multiplier bootstrap-t performs the best. It maintains a coverage rate consistent with the nominal level of 0.95 across variations in sample size and noise distribution. In general, methods with T standardization have a coverage rate closer to the nominal level than their counterparts with Z standardization, especially when sample size is small.

When the noise follows a Gaussian distribution, the nonparametric bootstrap-t method is overly conservative with small samples, yet aligns more with the nominal level as sample size increases. Conversely, when the noise follows a t distribution with 3 degrees of freedom ($t_{3}$), the nonparametric bootstrap-t remains excessively conservative and shows no improvement with larger samples.

Regarding runtime, as illustrated in Figure 3B, methods with Z standardization are faster across all sample sizes. They complete in less than 0.3 seconds for a single simulation instance involving 1,000 bootstrap iterations, which is approximately half the runtime required by T standardization. Within the same standardization, the three types of bootstrap methods have very similar runtime.

To understand the runtime difference between Z-standardization and T-standardization methods, we conducted a time-complexity analysis. Let K,N,B denote the number of voxels, sample size and the number of bootstrap samples, respectively. All bootstrap methods require computing the mean of each bootstrap sample, corresponding to a time-complexity of O(KNB) . With Z-standardization, the SD is computed once from the original data at a cost of O(KN) , which is negligible compared to the bootstrap mean computation. In contrast, T-standardization computes the SD separately for each bootstrap sample, adding an extra O(KNB) step. Thus, while both methods have the same asymptotic time-complexity of O(KNB) , T-standardization is computationally more expensive, approximately doubling the runtime due to additional matrix operations in bootstrap SD computation.

**Fig. 4.**
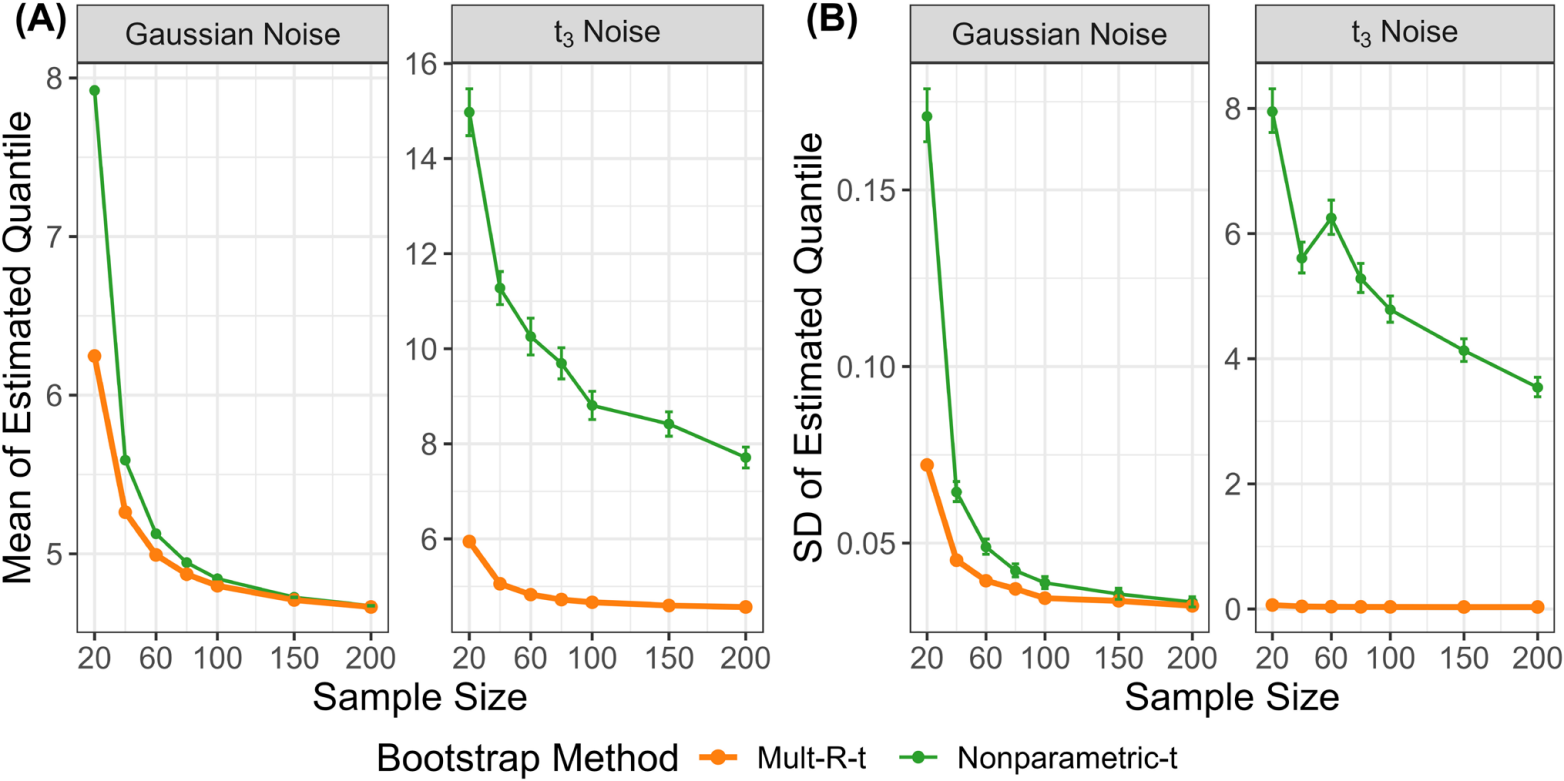
Results of 2D simulations on mean (A) and SD (B) of estimated SCB quantiles, under variations in sample size and noise distribution. Two bootstrap methods that achieved a good coverage rate are compared. A smaller mean quantile indicates a narrower (i.e., more precise) SCB and a smaller SD of quantiles indicates a more stable SCB. The error bars represent 95% confidence intervals (CIs). For the mean of estimated quantile, CI = $\text{mean}\pm 1.96\text{SD}\;/\;\sqrt{1000}$ . For the SD of the estimated quantile, CI = $\left[\text{SD}\sqrt{\frac{999}{\chi_{999,0.975}^{2}}},\text{ SD}\sqrt{\frac{999}{\chi_{999,0.025}^{2}}}\right]$, using the formula from Sheskin (2003). Some CIs are very narrow and may not be visible on the plot. Results for sample size of 10 are not plotted since several values are too large to display (e.g., under $t_{3}$ noise, mean = 33, SD = 13 for nonparametric-t; mean = 9, SD = 0.3 for Rademacher multiplier-t). The Rademacher multiplier-t gives a more precise and stable SCB than its competitor, the nonparametric-t under all scenarios.

Figure 4 presents the results for the mean and SD of estimated SCB quantiles, assessing precision and stability of each method. Only the two methods—Rademacher multiplier-t and nonparametric-t—that achieved coverage rates close to the nominal level are shown, since it is meaningless to consider precision and stability for methods with poor coverage. Under all scenarios, the Rademacher multiplier bootstrap-t gives a more precise and stable SCB than its main competitor, the nonparametric-t method.

**Fig. 5.**
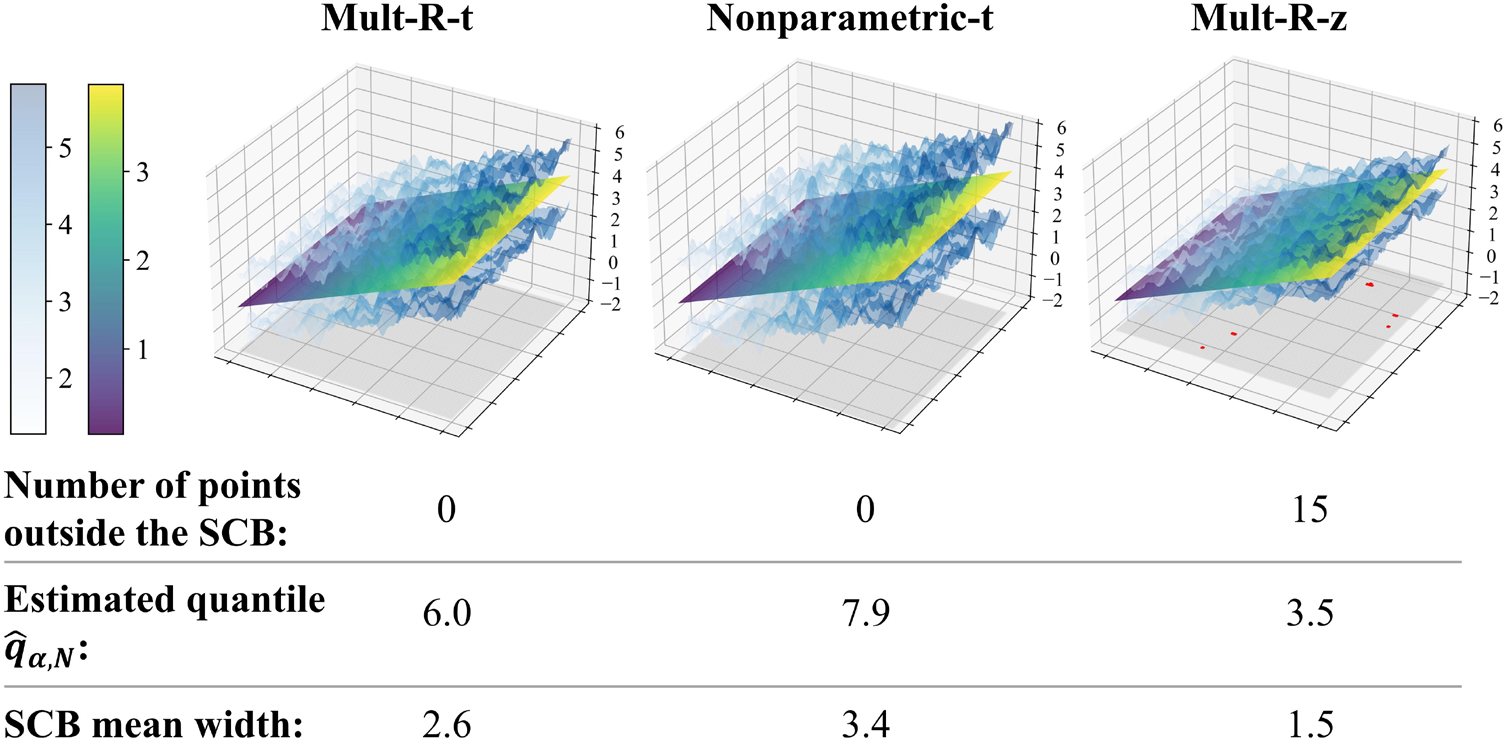
Example SCBs constructed by different bootstrap methods, under a simulated scenario with a 2D ramp-shaped signal of size 100×100 , sample size of 40, FWHM smoothing of 4 and SD of 1. The two blue surfaces represent the SCB of the 2D signal. The red dots indicate locations where the signal is not covered by the SCB. Of note, $\text{SCB}(s)={\hat{\mu }}_{N}\left(s\right)\pm {\hat{q}}_{\alpha ,N}\frac{{\hat{\sigma }}_{N}(s)}{\sqrt{N}}$. The SCB mean width was computed by averaging the band width across all 100×100 spatial locations. SCBs constructed by nonparametric-z, Mult-G-z, Mult-G-t were similar to those by Mult-R-z and are therefore omitted.

To illustrate the above findings, Figure 5 presents an example of SCBs constructed by different bootstrap methods, under a simulated scenario with a 2D ramp-shaped signal. Both nonparametric bootstrap-t and Rademacher multiplier-t yield SCBs that successfully cover the truth across all pixels; however, the former produces overly wide SCBs, reducing precision. In contrast, SCBs constructed by the remaining four bootstrap methods are too narrow and suffer from under-coverage.

To assess the impact of the number of bootstrap samples (denoted as B), we repeated the simulations using B∈{100,200,500,1000,2000} . Results on coverage (Supplementary Fig. S2.27) and mean of estimated quantiles (Supplementary Fig. S2.28) remain highly similar across different B. However, the SD of estimated quantiles decreases as B increases (Supplementary Fig. S2.29), indicating that the resulting SCBs become more stable. This is expected since a larger number of bootstrap samples reduces the variability of quantile estimates. In practice, using 1,000 bootstrap samples provides a good balance between stability and computational cost.

Previous results demonstrate the superior performance of Rademacher multiplier-t under Gaussian and $t_{3}$ noise. The findings under $t_{3}$ noise are especially relevant as it closely approximates the noise distribution of fMRI data (Davenport et al., 2023). It may also be of interest to consider skewed noise distributions, for which Rademacher multiplier-t is not specifically designed. To this end, we examined scenarios with skewed noise using $\chi^{2}$ distributions with varying degrees of freedom (df). Of note, the noise was centered and scaled to have mean 0 and SD of 10. A larger df corresponds to a more symmetric distribution and $\chi_{v}^{2}$ converges to the normal distribution as v→∞ . Under scenarios with large skewness (e.g., $\chi_{5}^{2}$), Rademacher multiplier-t shows mild to moderate undercoverage, though it still outperforms all other methods except for the nonparametric-t method (Supplementary Fig. S2.30). When the noise distribution is less skewed, the Rademacher multiplier bootstrap-t performs well, achieving a coverage rate close to the target level. Nonparametric-t performs similarly well in these settings, although it tends to overcover at small sample sizes. Overall, Rademacher multiplier-t demonstrates robustness under slight to moderate skewness, consistent with observations by Davidson and Flachaire (2008).

**Fig. 6.**
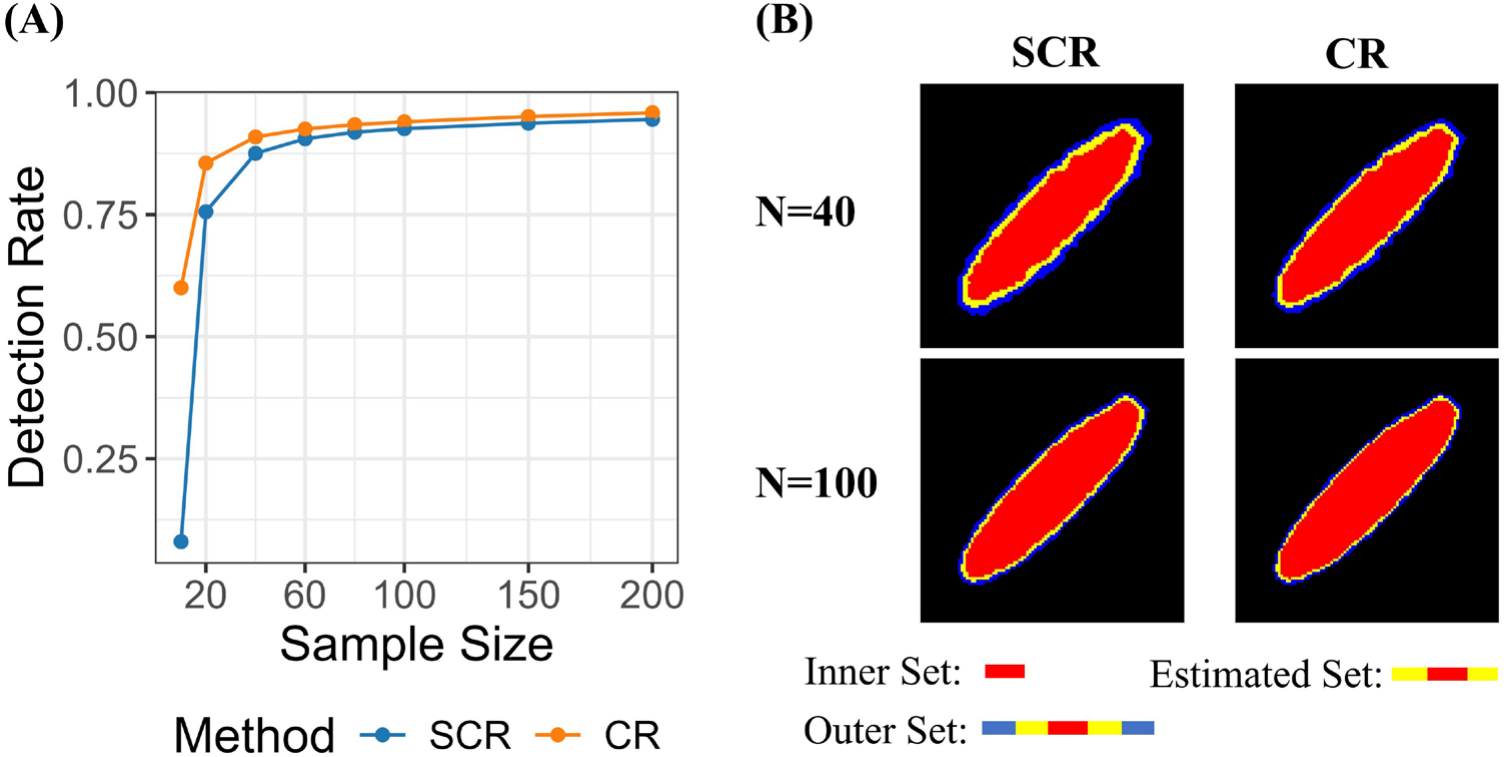
True positive detection rates (A) and example results (B) using the CR and SCR methods in scenarios with a 2D image of size 100×100 , ellipse-shaped signal, Gaussian noise, FWHM smoothing of 4 and SD of 1. Rademacher multiplier-t was used in both CR and SCR methods. The threshold was pre-specified at 2 to enable comparison between SCR and CR, although SCR does not require pre-specified thresholds since it remains valid for all thresholds. Results with $t_{3}$ noise are similar and are therefore omitted. (A) True positive detection rate is defined as the proportion of truly positive pixels in the inner set out of all pixels in the inner set, averaged across 1,000 simulations. (B) The red region, union of red and yellow region, union of red and yellow and blue region represent the inner set, estimated set, outer set, respectively.

Figure 6 compares the CR and SCR methods under a pre-specified threshold. As expected, the CR method achieves a higher true positive detection rate than the SCR method, indicating greater spatial precision. However, this difference diminishes when sample size N increases. When N≥40 , the detection rates are similar (e.g., 0.88 vs. 0.91 when N=40 ). Figure 6B presents an example of the CRs and SCRs, demonstrating that the two methods yield almost identical results when N is large.

### 4.2 3D validations

**Fig. 7.**
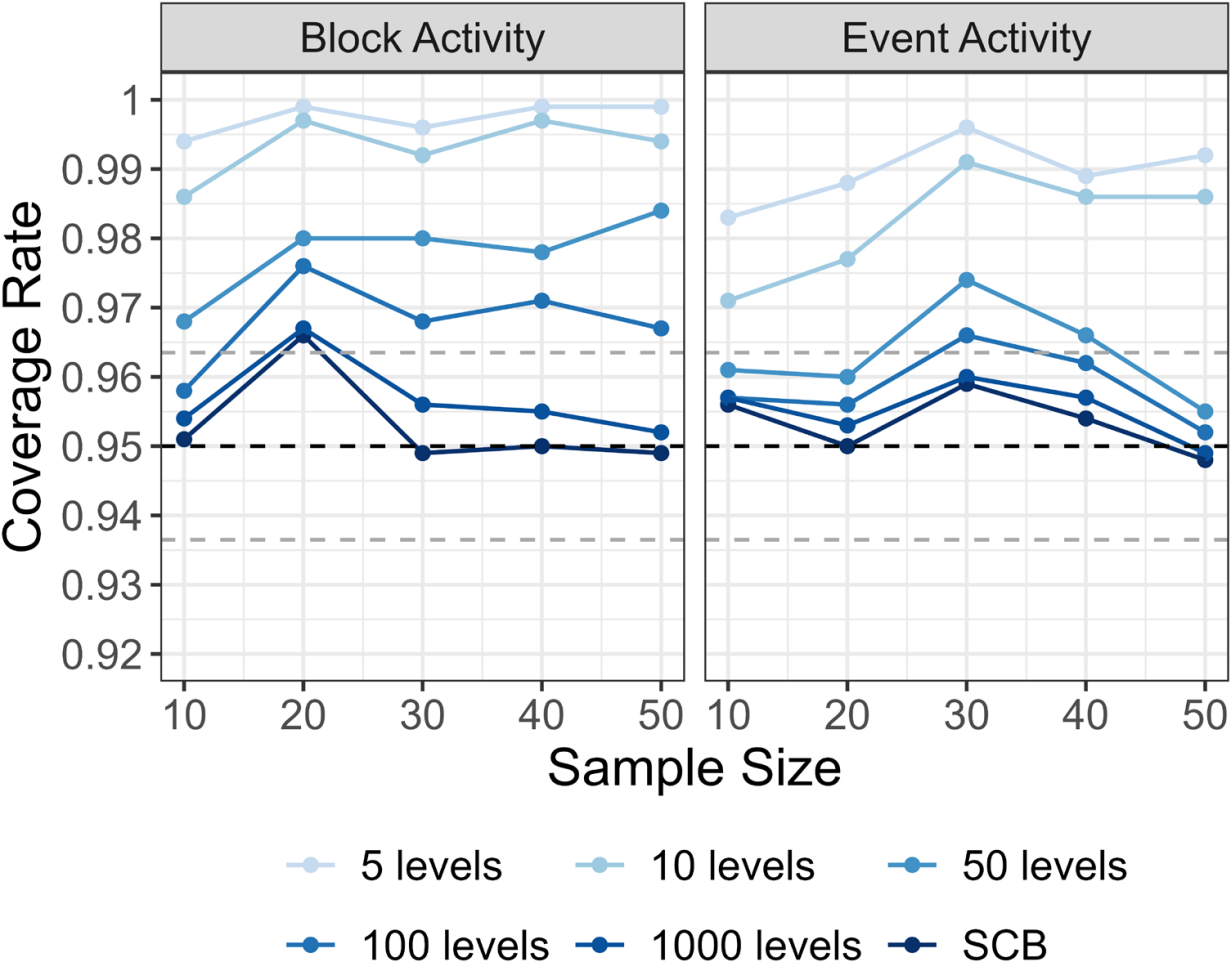
Coverage rate results of 3D validations with realistic fMRI data under variations in sample size and assumed activity paradigm. The confidence regions were constructed by inverting the SCBs obtained by the Rademacher multiplier bootstrap-t. The black dashed line represents the target coverage rate of 0.95. The two gray dashed lines capture the uncertainty due to simulation and correspond to $0.95\pm 1.96\times \sqrt{0.95(1-0.95)\;/\;1000}$ . The coverage rate of the SCB is close to the target level. Of note, the confidence bounds around 0.95 were calculated assuming independent simulations for simplicity, while in reality, these simulations are likely positively correlated due to overlapping samples across simulations. Therefore, the actual bounds are slightly wider. This explains why under the block activity paradigm, the SCB coverage slightly exceeds the upper bound at sample size of 20.

We conducted 3D validations using the SCR method with the Rademacher multiplier bootstrap-t, which achieved the target SCB coverage rate in previous 2D simulations. As depicted in Figure 7, the coverage rates of the SCBs closely align with the nominal level of 0.95, independent of the sample size and assumed activity paradigm, validating the use of the Rademacher multiplier bootstrap-t for SCB construction in realistic fMRI data. Regarding the resulting confidence regions, as expected, their coverage rates decrease and converge to the nominal level as the number of considered thresholds increases.

### 4.3 Application to task fMRI volume and surface data

**Fig. 8.**
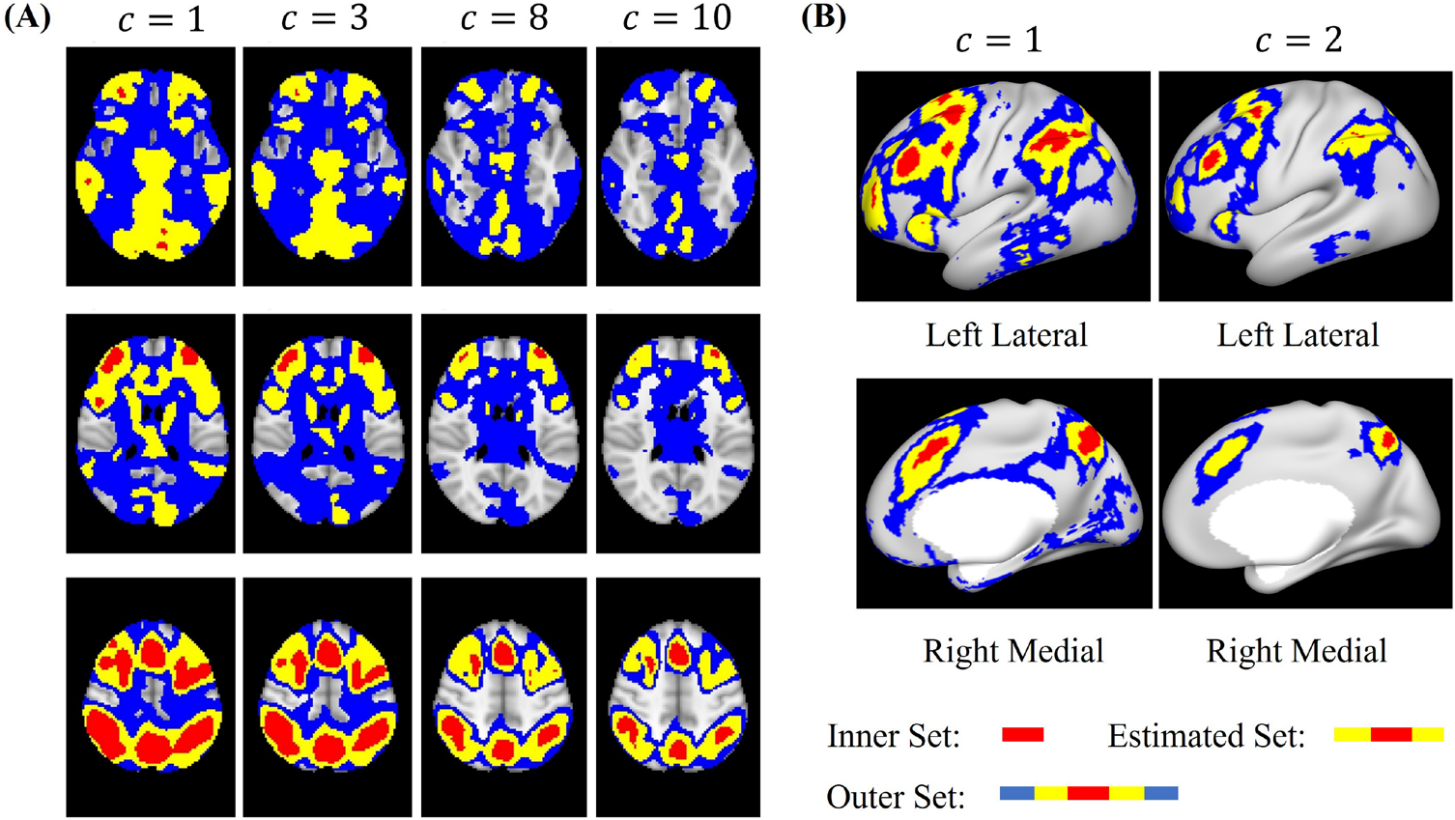
Confidence region results of fMRI volume data (A) and surface data (B) obtained during a working memory task. The red region, union of red and yellow region, union of red and yellow and blue region represent the inner set, estimated set, outer set, respectively. Each column displays the results for a particular threshold c by showing three distinct slices of the 3D brain in (A) or by showing the left and right hemispheres in (B). For example, the second column panel of (A) shows the result for brain regions with at least 3% BOLD change.

**Fig. 9.**
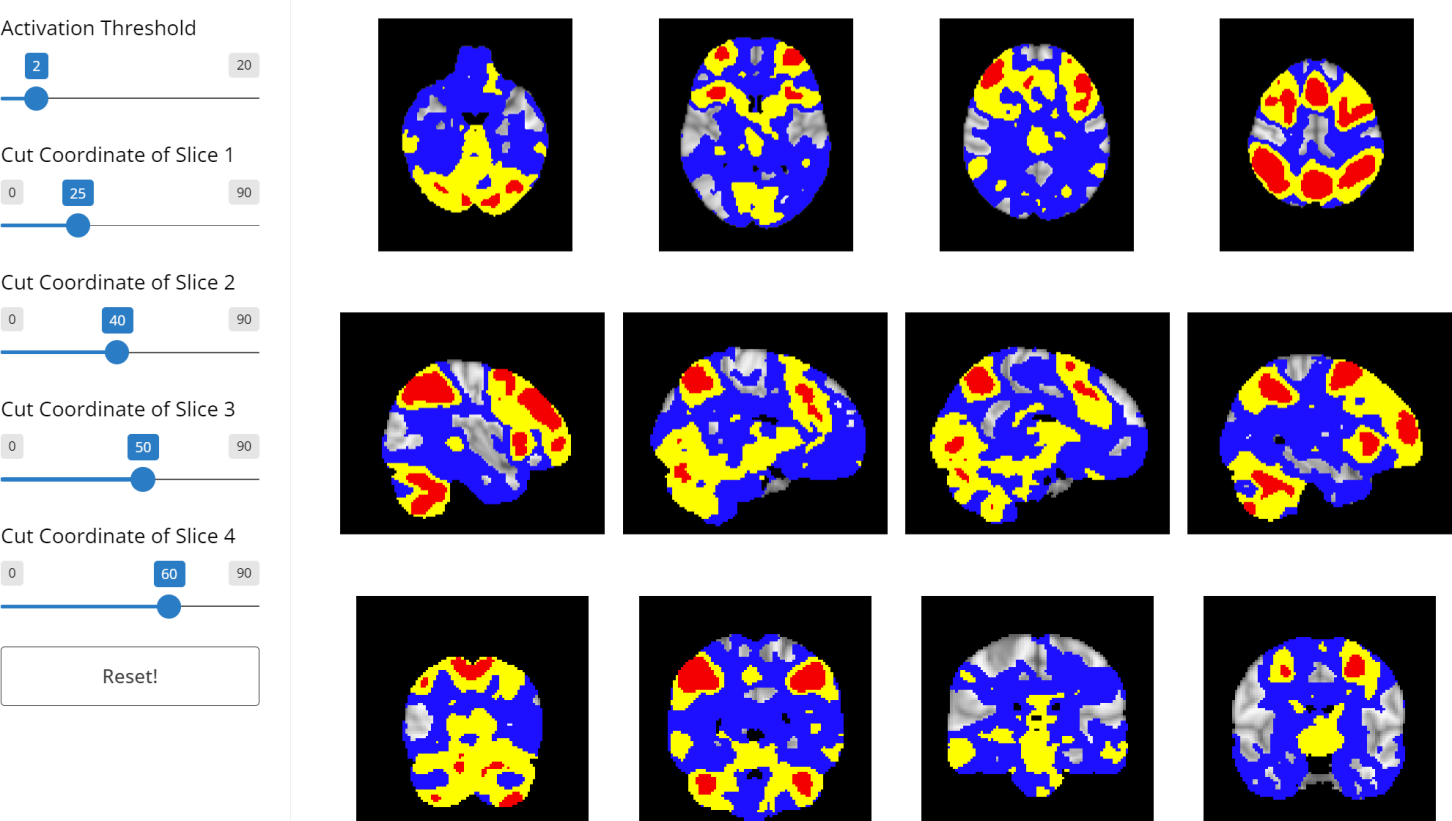
A demonstration of the interactive visualization tool for volume data analysis. This tool allows users to view the results of the confidence regions and estimated excursion sets as they change the activation threshold and the coordinates of four slices. Each column corresponds to a particular slice at a given coordinate. Each row corresponds to a particular direction of the slice: axial, sagittal, and coronal, listed from top to bottom.

**Fig. 10.**
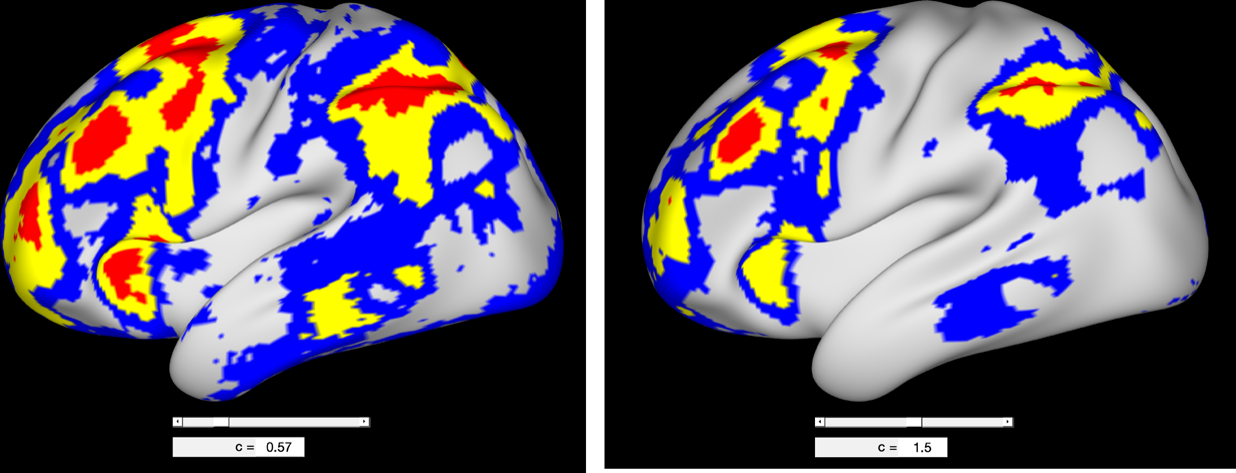
A demonstration of the interactive visualization tool for surface data analysis. This tool allows users to view the results of the confidence regions and estimated excursion sets as they change the activation threshold. In the example two thresholds, c=0.57 and 1.5 are shown (which are in units of % BOLD change).

The SCR method with the Rademacher multiplier bootstrap-t was applied to both the fMRI volume and surface data from HCP, collected during a working memory task. The results are presented in Figure 8A for volume data and Figure 8B for surface data. Demonstrations of interactive apps to visualize the results as users adjust the activation threshold are provided in Figures 9 and 10. Results with additional thresholds and slices in different directions are provided in the Supplementary File (Section S1). The results demonstrate that fronto-parietal brain regions were activated during the working memory task, consistent with prior studies (Chai et al., 2018; Engström et al., 2015).

In both analyses, the activation thresholds selected for presentation were those that yielded the most informative and interesting results after exploring a range of thresholds. A major advantage of this method is its capacity to provide valid inference at all potential thresholds, offering great flexibility. For example, with the second column in Figure 8A, we can conclude with at least 95% confidence that the brain region within the red area has an activation of at least 3% BOLD change. Similar conclusions can be made for all the other thresholds considered. Interactive apps to visualize the results as users adjust the activation threshold are demonstrated in Figures 9 and 10.

**Fig. 11.**
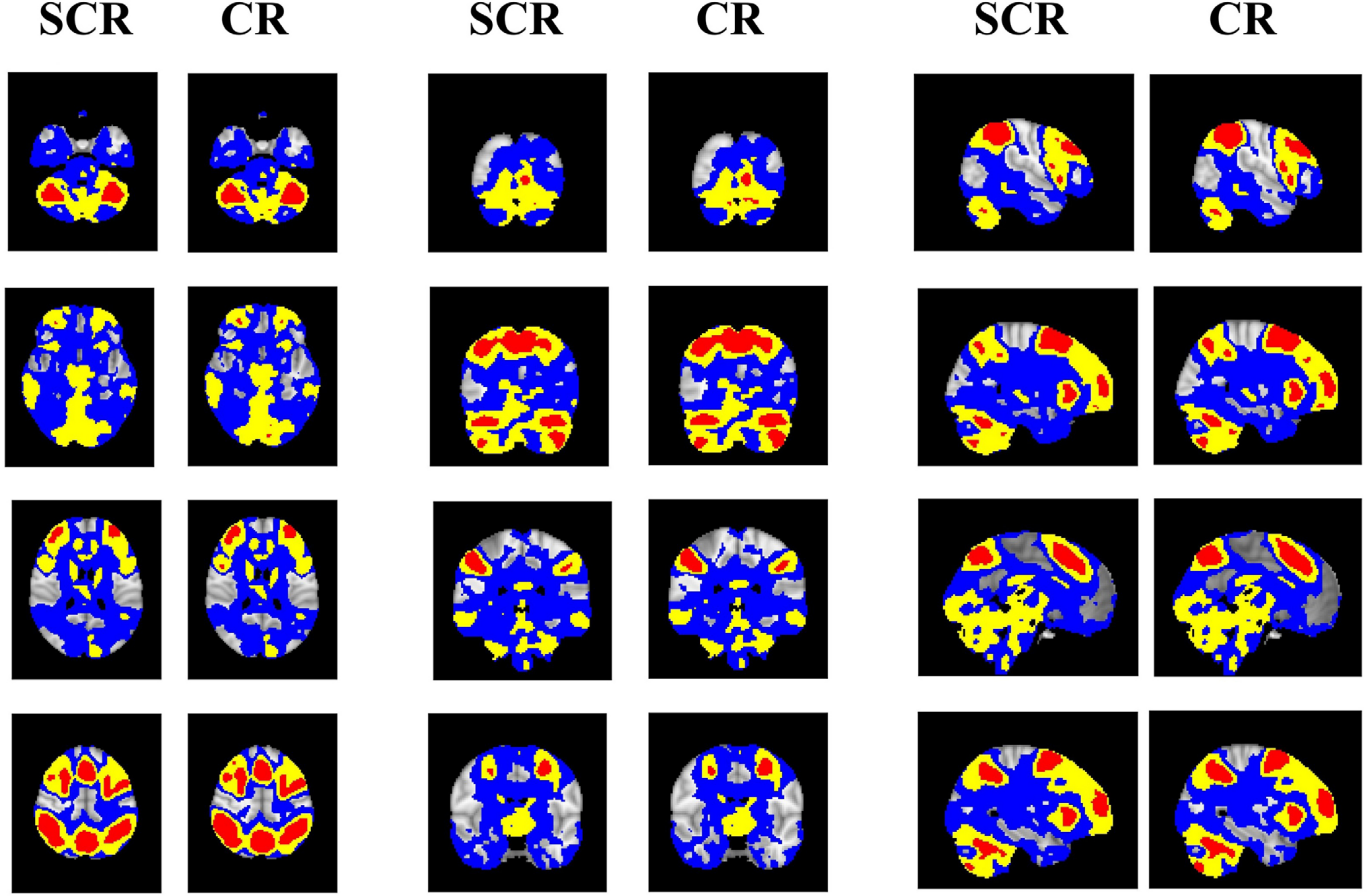
Analysis results of fMRI volume data from 78 subjects using CR and SCR methods, displayed in axial, sagittal, and coronal slices. The threshold was pre-specified at 3 to enable comparison between SCR and CR, although SCR does not require pre-specified thresholds since it remains valid for all thresholds. The red region, union of red and yellow region, union of red and yellow and blue region represent the inner set, estimated set, outer set, respectively. Each row represents a particular slice.

If users have a pre-specified threshold, the CR method from Bowring et al. (2019) can be applied. Figure 11 presents the CR results alongside our SCR results at a threshold of 3% BOLD change, showing that the two methods give similar results. However, our SCR method provides great flexibility, enabling users to perform inference across all thresholds simultaneously while maintaining statistical rigor.

## 5 Discussion

In this study, we extended the SCR method in Ren et al. (2024) to the neuroimaging setting. We evaluated six bootstrap approaches for SCB construction using 2D simulations. The Rademacher multiplier bootstrap with T standardization performed the best, achieving a coverage rate close to the nominal level with sample sizes as low as 10. We further validated this method using real resting-state 3D fMRI data, a technique that has become the gold standard, by creating realistic noise that reflects the non-Gaussian and non-stationary structure of fMRI data. Our applications to real task fMRI volume data and surface data showcase the utility of this method in neuroimaging. Moreover, we have developed software packages which implement this method and are equipped with visualization tools designed for fMRI. In conclusion, we confirm the validity of this method with the Rademacher multiplier bootstrap-t and advocate for its broader application in fMRI studies for localizing activated brain regions.

A key advantage of SCRs is that they provide valid inference simultaneously across all activation thresholds. This enables researchers to explore the data more flexibly and choose the thresholds which provide the most interesting results, without concerns about multiple comparison issues over thresholds. We have developed interactive tools for both volume and surface data analyses, allowing users to visualize the activated brain regions as they adjust the threshold. Another strength of our method is that it does not assume stationarity, a particular correlation structure or distribution on the noise field and has demonstrated robustness across various settings considered in our simulations. This reduces bias from model misspecification compared to other methods such as classical implementations based on random field theory (Worsley et al., 1996, 2004), which have been shown to perform poorly in fMRI in some settings due to the non-stationarity (Eklund et al., 2016) and high levels of non-Gaussianity (Davenport et al., 2023).

Our 2D simulations assessed six bootstrap methods on coverage rate and runtime. Regarding coverage rate, the superior performance of the Rademacher multiplier bootstrap-t aligns with previous studies which considered simpler 1D or Gaussian scenarios (Bowring et al., 2019; Telschow & Schwartzman, 2022). The Rademacher multiplier bootstrap-t has been mathematically proved to achieve nominal coverage rate asymptotically in functional SCB (Chang et al., 2017) and has demonstrated superior performance in other contexts. For example, Davidson and Flachaire (2008) investigated bootstrap methods for hypothesis testing in linear regression and recommended Rademacher multipliers. Additionally, Liu (1988) established that if the population follows a symmetric distribution, then Edgeworth expansions of the target statistic distribution and the wild-bootstrapped version can be best matched under the condition that the first four moments of the multiplier are 0, 1, 0, 1, with Rademacher distribution being the only one satisfying this condition, which explains its superior performance over other multipliers. Although nonparametric-t also achieved good coverage rates, it produced wider and less stable SCBs. This is because this method may be numerically unstable, leading to excessively wide confidence bands (Diciccio & Romano, 1988). Furthermore, we observed that methods with T standardization outperform their counterparts with Z standardization, especially at small sample sizes ≤60 , consistent with results in Telschow and Schwartzman (2022). This finding is not surprising since T standardization mimics the construction of the standard bootstrap-t interval, which has been mathematically proved to be accurate (Diciccio & Romano, 1988; Hall, 1988).

Regarding runtime, we found bootstrap approaches with T standardization had roughly twice the runtime of those with Z standardization, regardless of the bootstrap type. This is consistent with our time-complexity analysis, which shows that both standardization types have same asymptotic time-complexity, but T standardization has a larger constant factor since it calculates the SD of each bootstrap sample separately, whereas Z standardization only calculates the SD of the original sample once. Nonetheless, with 1,000 bootstrap samples, image size of 100×100 and sample size of 200, methods with T standardization completed within 0.6 seconds on a regular laptop, suggesting runtime concerns are minimal. Considering both aspects of coverage rate and runtime, we recommend the use of the Rademacher multiplier bootstrap-t.

Our 3D resting-state validations showed that SCRs using the Rademacher multiplier bootstrap-t control the coverage rate at or above the nominal level in realistic fMRI data. As the number of thresholds increases, the coverage rate of the confidence regions approaches that of the SCBs from above. This occurs because the probability of coverage at a finite number of thresholds is always greater than for all thresholds, with equality in the limit. This allows the user to choose the threshold, even data driven, without worrying about incurring additional error.

Using our approach we explored the brain regions which are activated during a working memory task in both volume and surface data. The results are in line with previous research that associates working memory with fronto-parietal brain regions (Chai et al., 2018; Engström et al., 2015). However, prior results were obtained using hypothesis testing under the null of no activation, without providing spatial uncertainty (see, e.g., figure 3 in Engström et al., 2015). In contrast, our method shows spatial uncertainty and captures the strength of the activation in interpretable units of %BOLD change.

Since our method enjoys valid inference for all thresholds simultaneously, it is conservative when users have specific predetermined thresholds of interest. In that case, the CR method in Bowring et al. (2019) for a single threshold and the method in Telschow et al. (2023) for a range of thresholds may offer greater spatial precision. Nonetheless, based on our simulations and real-data application, when sample size is moderate (e.g., 78 in our application), the loss in spatial precision of the more flexible SCR method is minimal. Moreover, with larger sample sizes, SCR and CR produce nearly identical results, highlighting the versatility and practical value of the SCR method.

Our work can be extended in the following directions. First, our implementation of the method focuses on a second-level analysis to estimate population mean, where it is reasonable to assume the contrast images from different individuals are i.i.d. In first-level analyses, where the time series of the BOLD response during an fMRI experiment is analyzed, the i.i.d. assumption is violated. In such cases, SCB construction methods tailored for time series, for instance using the block bootstrap (Politis, 2003) to estimate the quantile, could be used. Once a valid SCB is established, SCRs can be constructed similarly by inverting the SCB. Second, non-bootstrap methods for constructing SCBs could be considered, such as those based on the functional central limit theorem (Degras, 2011) or the Gaussian kinematic formula (Telschow & Davenport, 2023; Telschow & Schwartzman, 2022). Third, extensions to non-linear test statistics could also be considered. One approach involves bootstrapping delta residuals, which are transformed residuals that asymptotically have the same covariance structure as the limit process involved in the functional delta method (Telschow et al., 2022).

## Supplementary Material

Supplementary Material

## Data Availability

Data and code are available at https://github.com/JiyueQin/SimuInf. The Human Connectome Project data can be provided upon request after users sign the data use agreement required by HCP, as instructed in the ReadMe file of the above GitHub link.
