# Supplementary material for: Simultaneous confidence regions for image excursion sets: A validation study with applications in fMRI
Source: Imaging Neurosci (Camb). 2025 Dec 5;3:IMAG.a.1044. doi: 10.1162/IMAG.a.1044 (PMC13288502; doi:10.1162/IMAG.a.1044)
Supplement: Supplementary Material [file IMAG.a.1044_supp.pdf]

Supplementary Results

Contents

1 Results of Task fMRI Volume Data Analysis **2**

1.1 Axial Slices . . . 2

1.2 Sagittal Slices . . . 3

1.3 Coronal Slices . . . 4

2 2D Simulation Results **5**

2.1 Coverage Rate . . . 5

2.2 Runtime . . . 13

2.3 Precision . . . 15

2.4 Stability . . . 23

2.5 Impact of Different Numbers of Bootstrap Samples . . . 31

2.6 Skewed Noise Distributions . . . 34

1 Results of Task fMRI Volume Data Analysis

1.1 Axial Slices

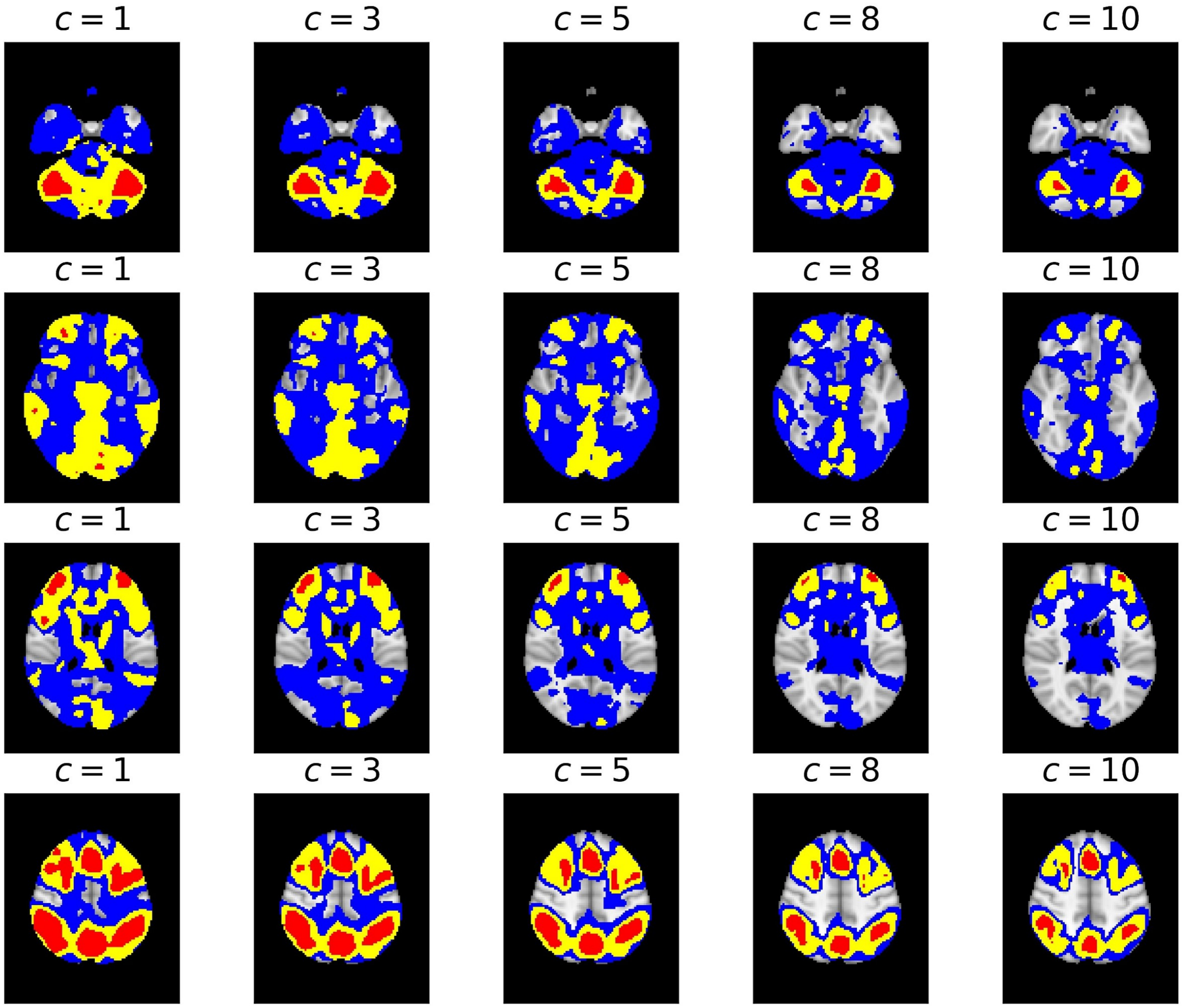

Figure 1.1: Confidence region results of fMRI volume data obtained, displayed in axial slices. The red region, union of red and yellow region, union of red and yellow and blue region represent the inner set, estimated set, outer set, respectively. Each column represents a particular activation threshold c and each row represents a particular axial slice.

1.2 Sagittal Slices

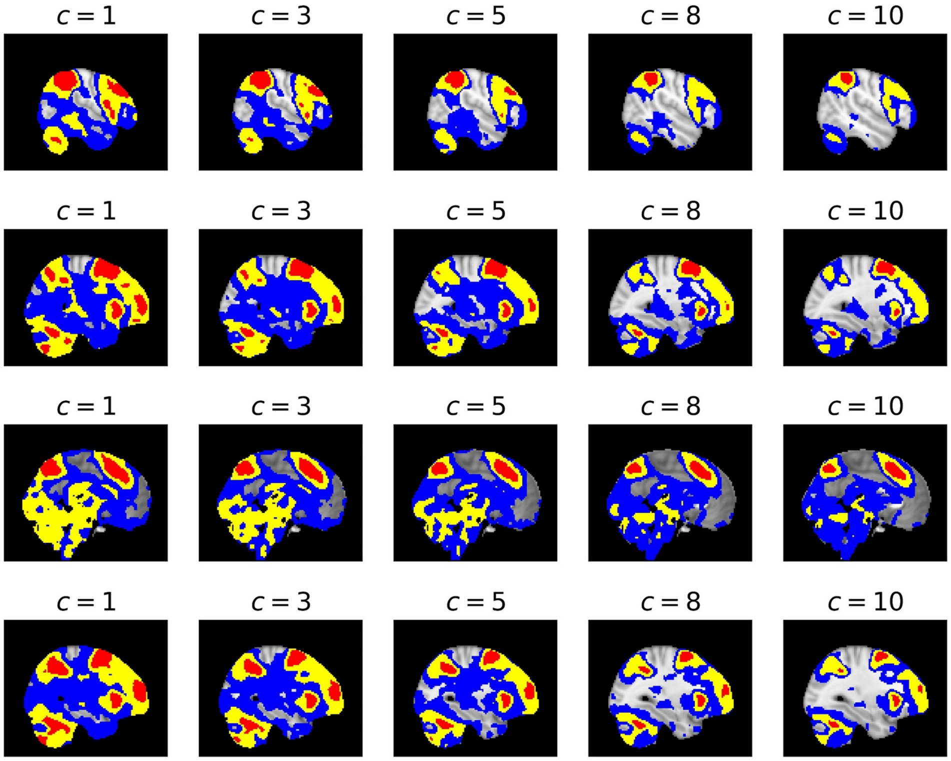

Figure 1.2: Confidence region results of fMRI volume data, displayed in sagittal slices. The red region, union of red and yellow region, union of red and yellow and blue region represent the inner set, estimated set, outer set, respectively. Each column represents a particular activation threshold c and each row represents a particular sagittal slice.

1.3 Coronal Slices

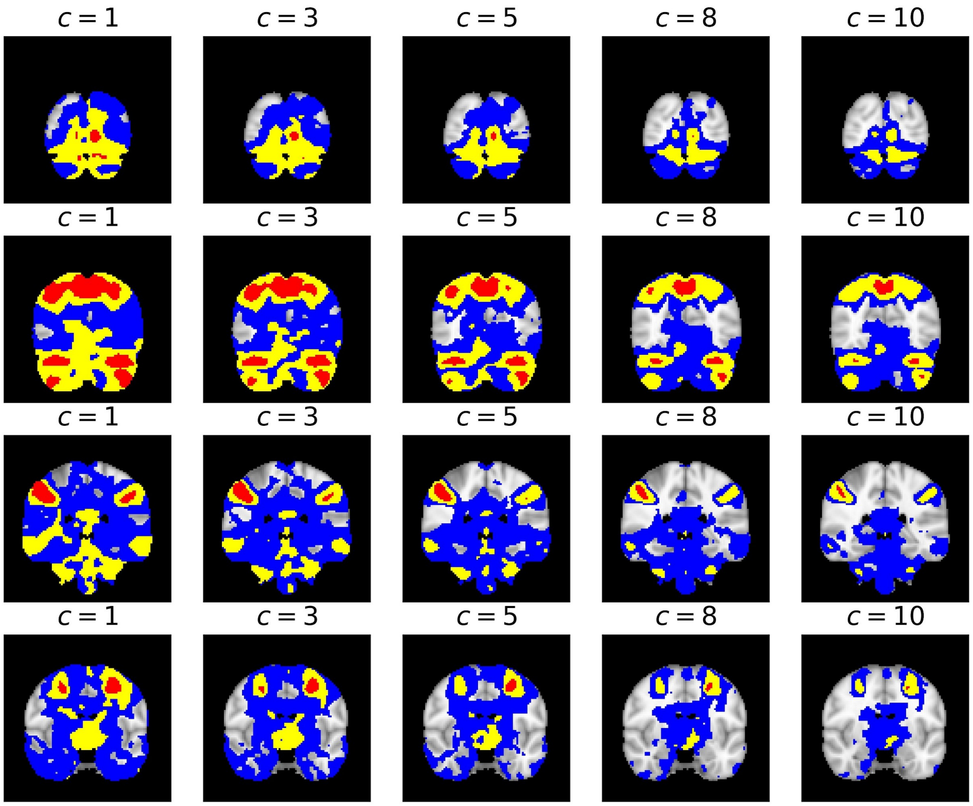

Figure 1.3: Confidence region results of fMRI volume data, displayed in coronal slices. The red region, union of red and yellow region, union of red and yellow and blue region represent the inner set, estimated set, outer set, respectively. Each column represents a particular activation threshold c and each row represents a particular coronal slice.

2 2D Simulation Results

2.1 Coverage Rate

Results of coverage rate in all simulated scenarios:

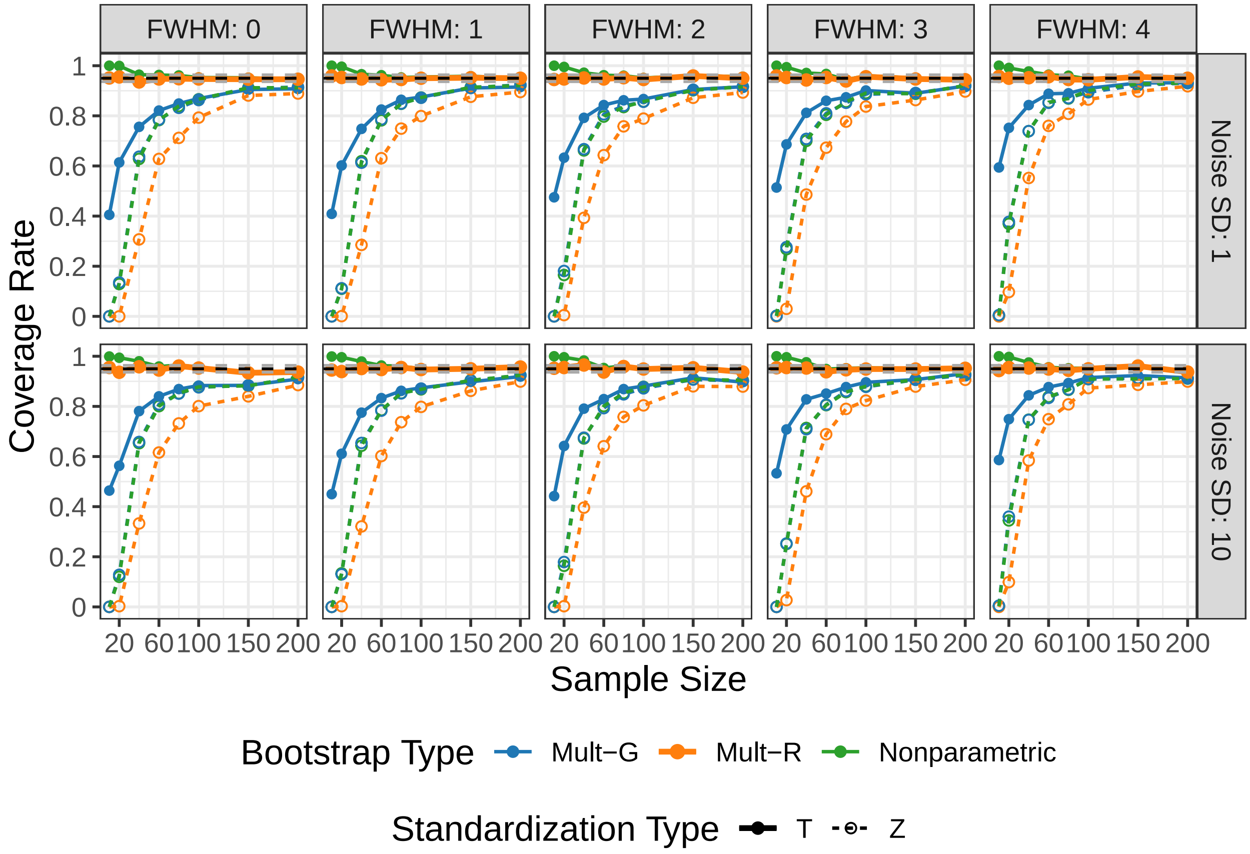

Figure 2.1: Coverage results in scenarios with ellipse shape, gaussian noise distribution and image size of 50×50. Six bootstrap methods (3 bootstrap types $\times$ 2 standardization types) were evaluated. The black dashed line indicates the target coverage rate of 0.95. The two gray dashed lines capture the uncertainty due to simulation and correspond to $0.95 \pm 1.96 \times \sqrt{0.95(1-0.95)/1000}$.

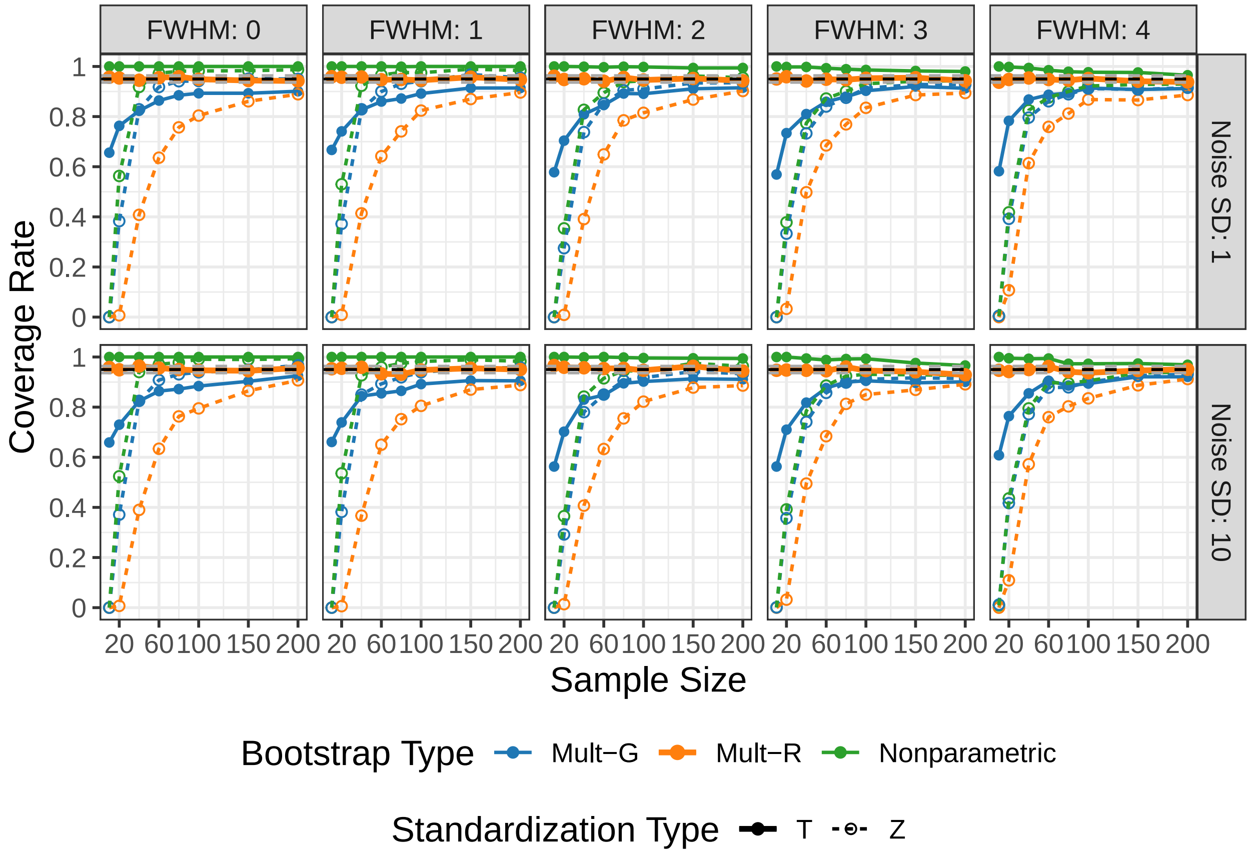

Figure 2.2: Coverage results in scenarios with ellipse shape, t noise distribution and image size of 50 × 50. Six bootstrap methods (3 bootstrap types × 2 standardization types) were evaluated. The black dashed line indicates the target coverage rate of 0.95. The two gray dashed lines capture the uncertainty due to simulation and correspond to $0.95 \pm 1.96 \times \sqrt{0.95(1-0.95)/1000}$.

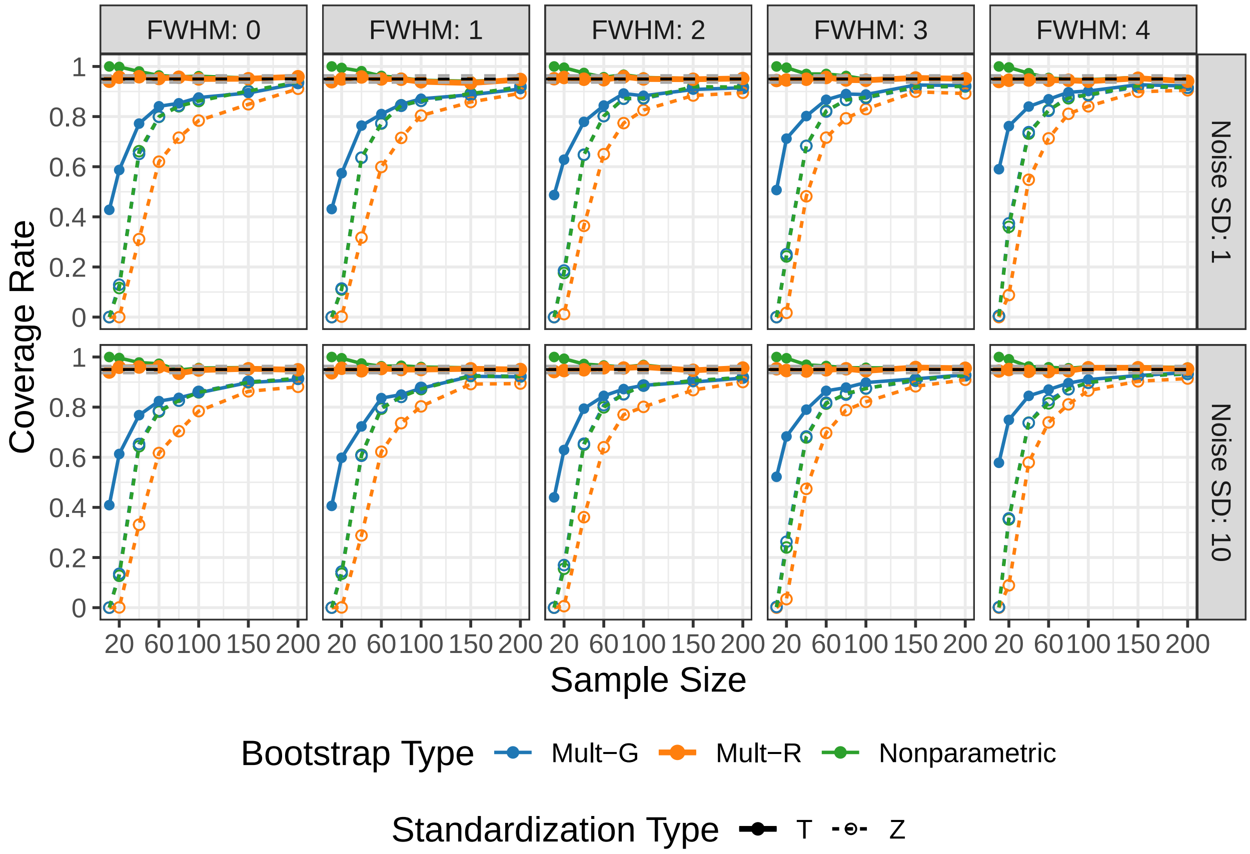

Figure 2.3: Coverage results in scenarios with ramp shape, gaussian noise distribution and image size of 50×50. Six bootstrap methods (3 bootstrap types $\times$ 2 standardization types) were evaluated. The black dashed line indicates the target coverage rate of 0.95. The two gray dashed lines capture the uncertainty due to simulation and correspond to $0.95 \pm 1.96 \times \sqrt{0.95(1-0.95)/1000}$.

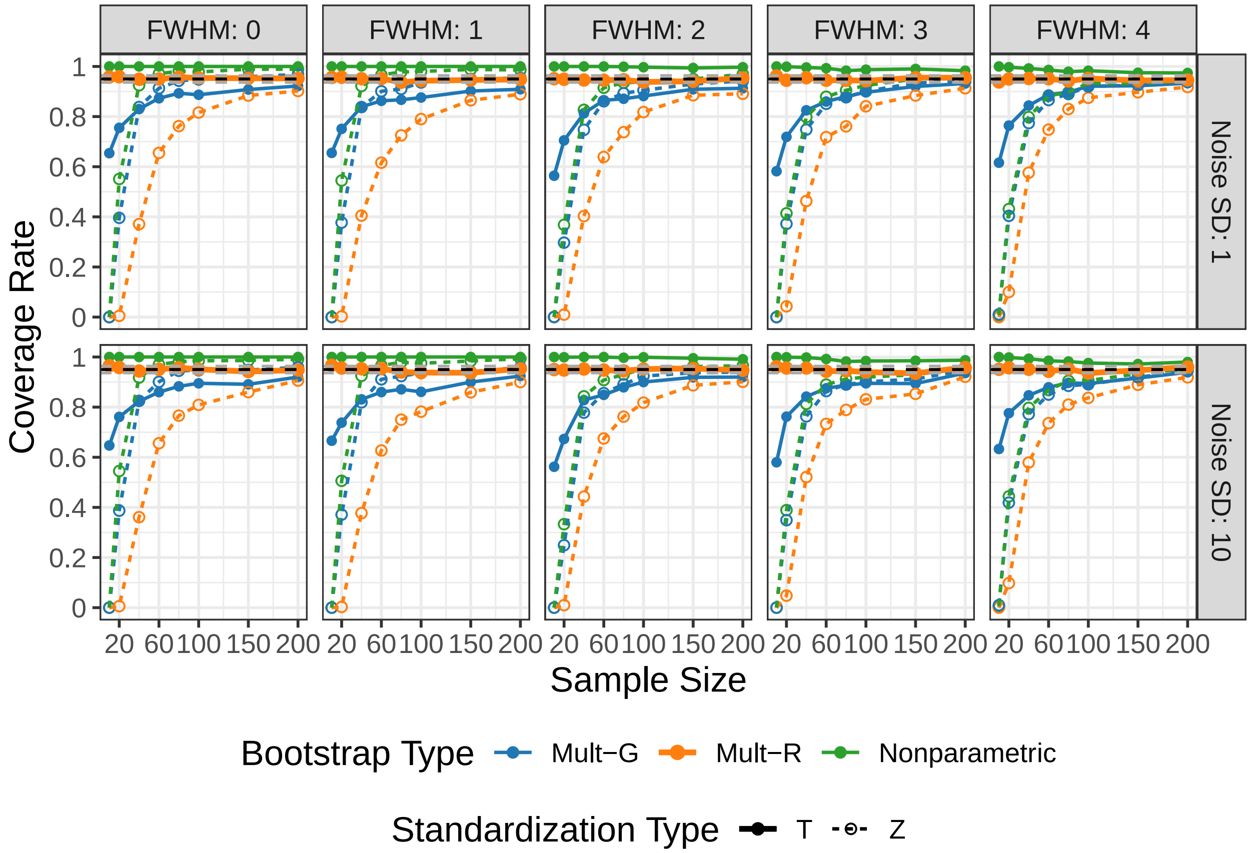

Figure 2.4: Coverage results in scenarios with ramp shape, t noise distribution and image size of 50×50. Six bootstrap methods (3 bootstrap types $\times$ 2 standardization types) were evaluated. The black dashed line indicates the target coverage rate of 0.95. The two gray dashed lines capture the uncertainty due to simulation and correspond to $0.95 \pm 1.96 \times \sqrt{0.95(1-0.95)/1000}$.

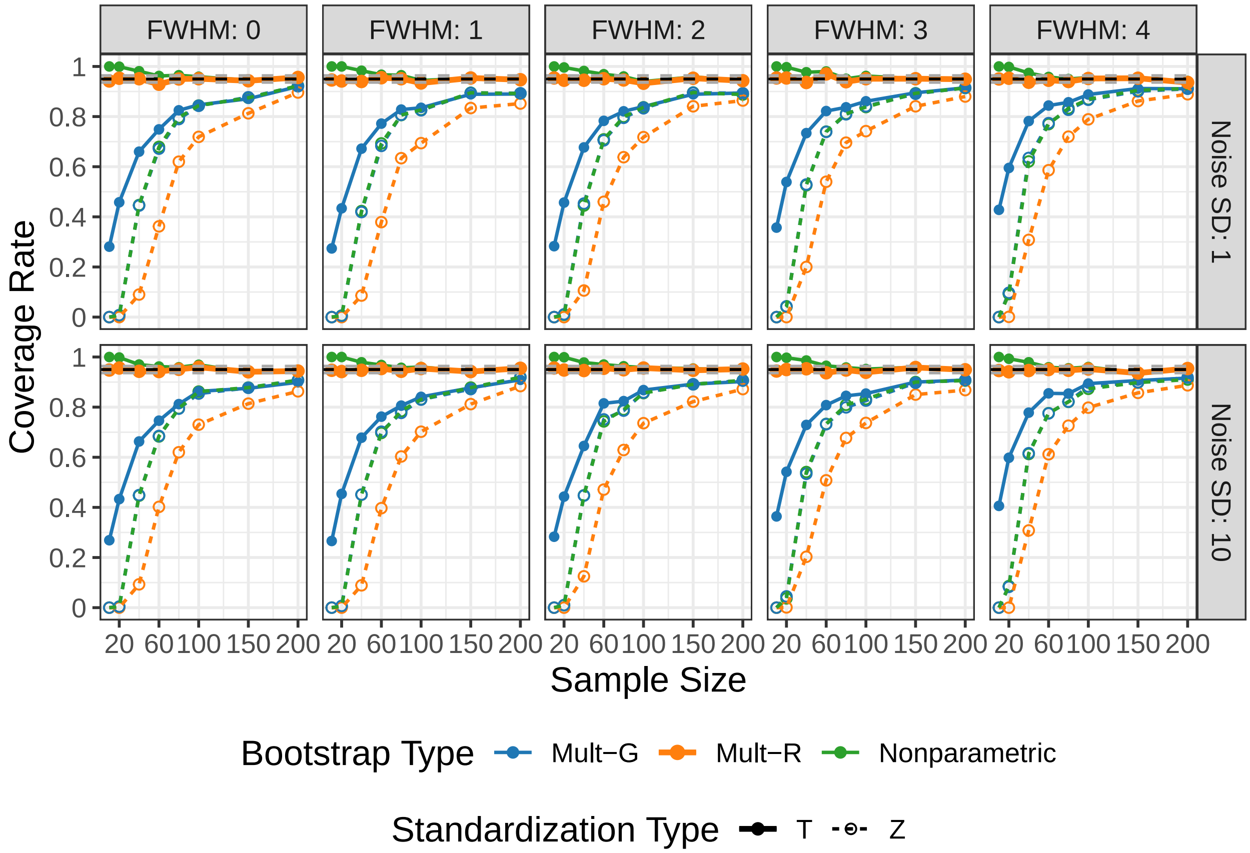

Figure 2.5: Coverage results in scenarios with ellipse shape, gaussian noise distribution and image size of 100×100. Six bootstrap methods (3 bootstrap types $\times$ 2 standardization types) were evaluated. The black dashed line indicates the target coverage rate of 0.95. The two gray dashed lines capture the uncertainty due to simulation and correspond to $0.95 \pm 1.96 \times \sqrt{0.95(1-0.95)/1000}$.

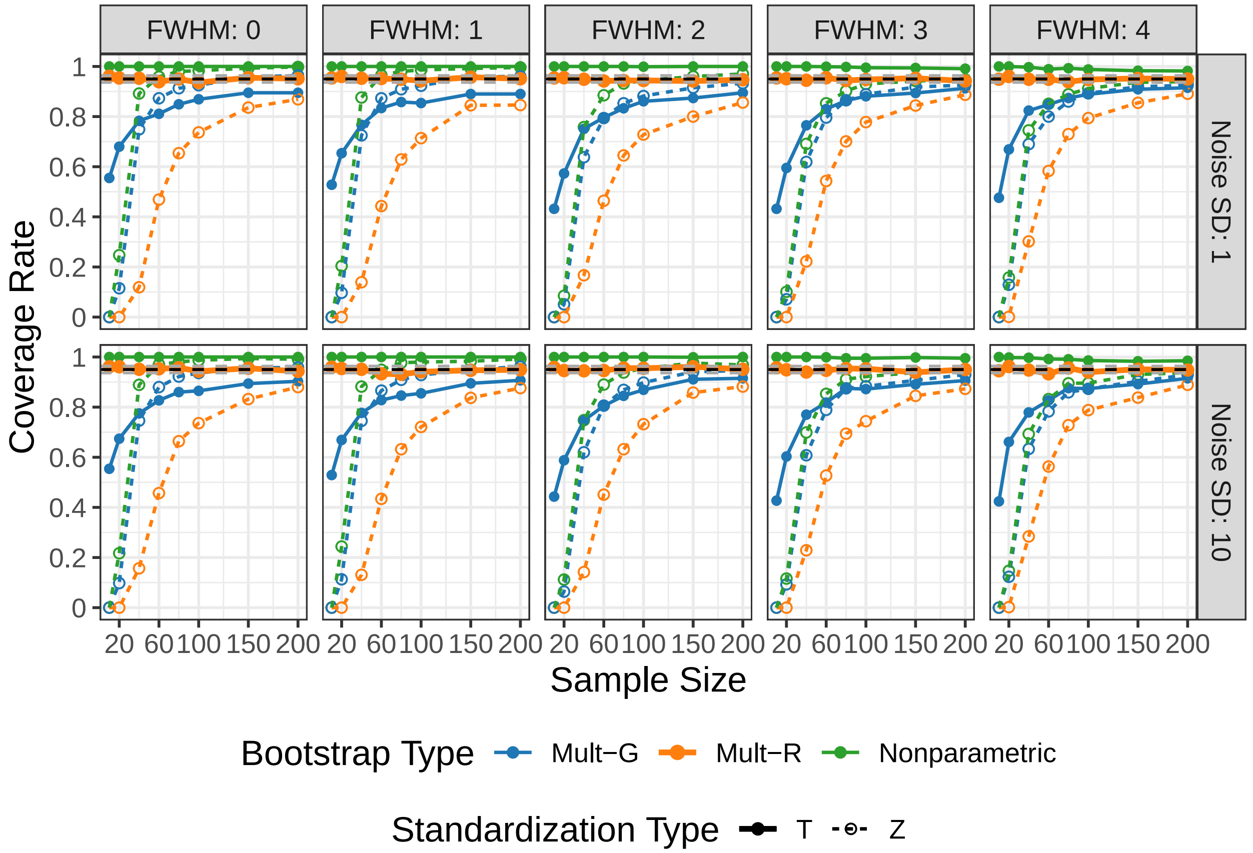

Figure 2.6: Coverage results in scenarios with ellipse shape, t noise distribution and image size of 100 × 100. Six bootstrap methods (3 bootstrap types × 2 standardization types) were evaluated. The black dashed line indicates the target coverage rate of 0.95. The two gray dashed lines capture the uncertainty due to simulation and correspond to $0.95 \pm 1.96 \times \sqrt{0.95(1-0.95)/1000}$.

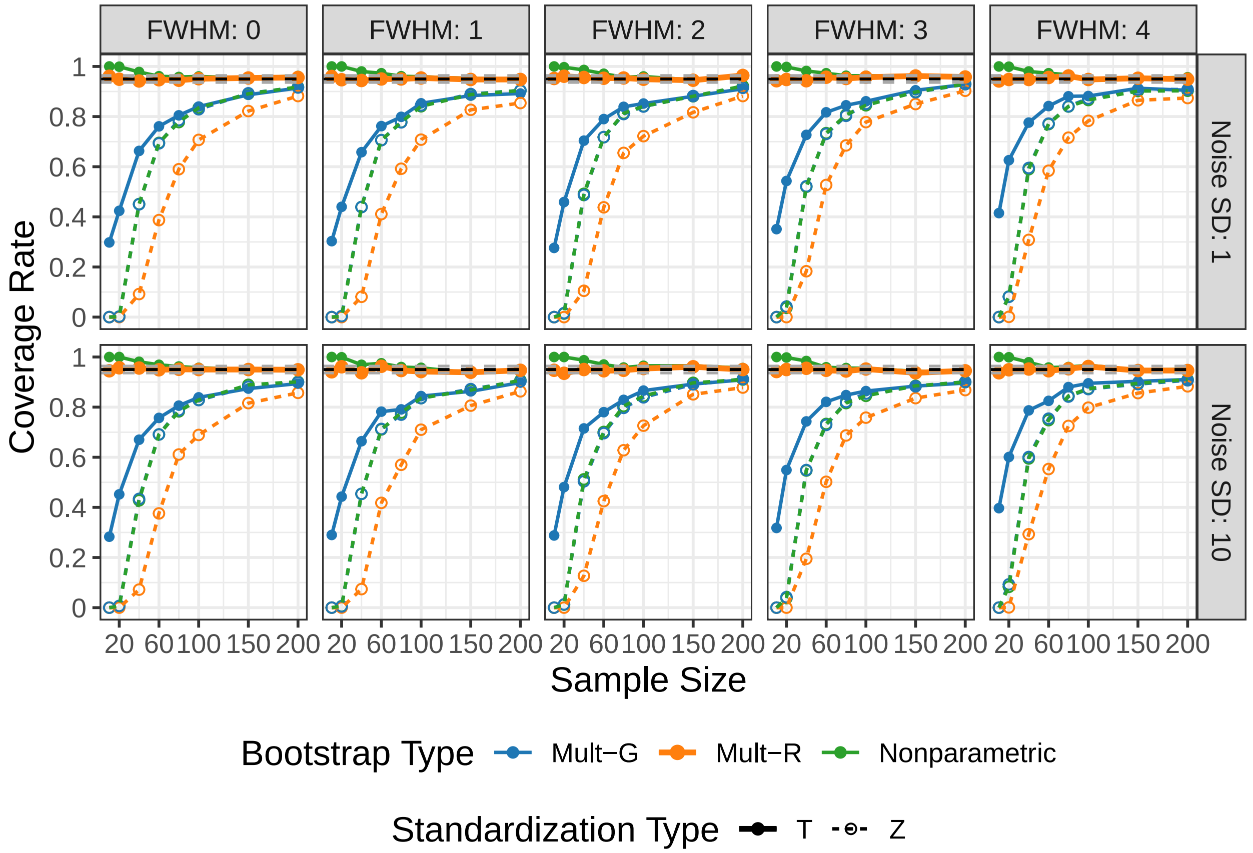

Figure 2.7: Coverage results in scenarios with ramp shape, gaussian noise distribution and image size of 100×100. Six bootstrap methods (3 bootstrap types $\times$ 2 standardization types) were evaluated. The black dashed line indicates the target coverage rate of 0.95. The two gray dashed lines capture the uncertainty due to simulation and correspond to $0.95 \pm 1.96 \times \sqrt{0.95(1-0.95)/1000}$.

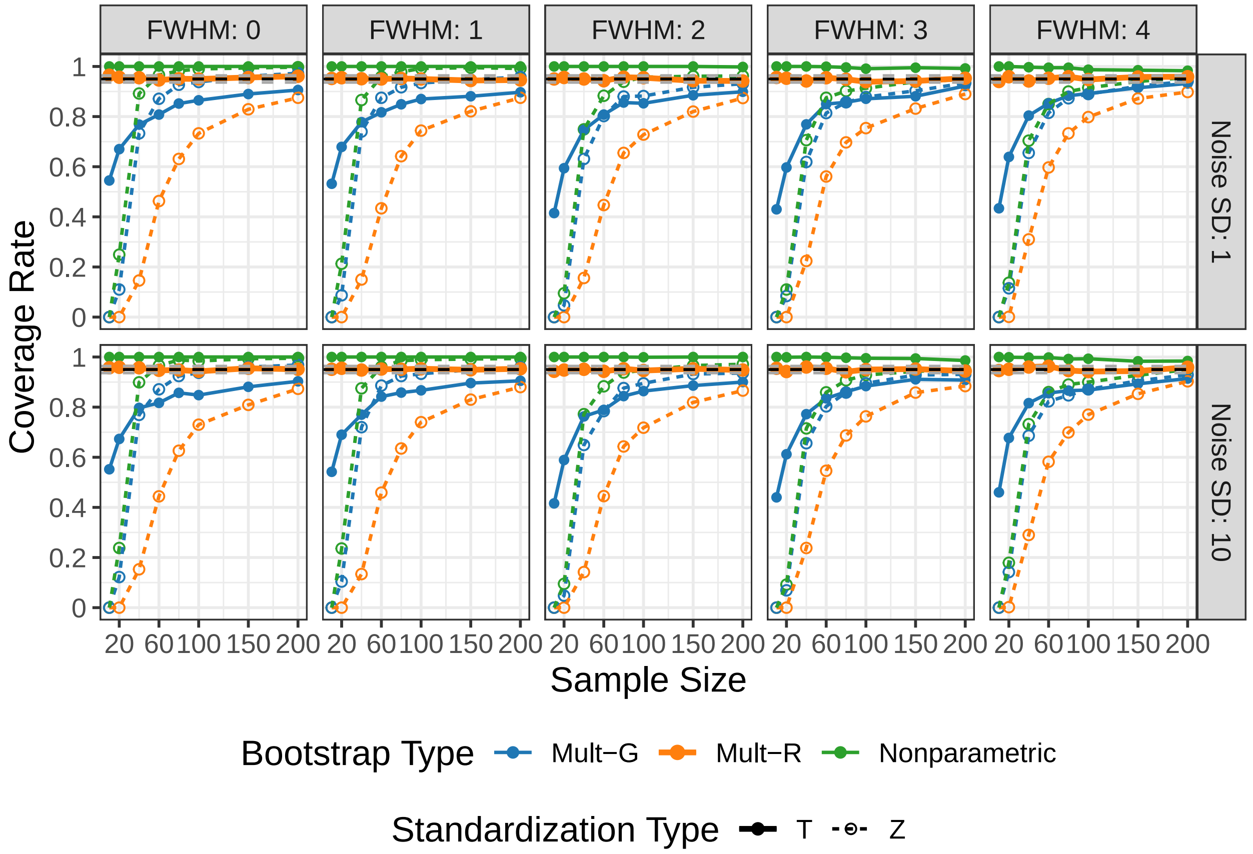

Figure 2.8: Coverage results in scenarios with ramp shape, t noise distribution and image size of 100×100. Six bootstrap methods (3 bootstrap types $\times$ 2 standardization types) were evaluated. The black dashed line indicates the target coverage rate of 0.95. The two gray dashed lines capture the uncertainty due to simulation and correspond to $0.95 \pm 1.96 \times \sqrt{0.95(1-0.95)/1000}$.

2.2 Runtime

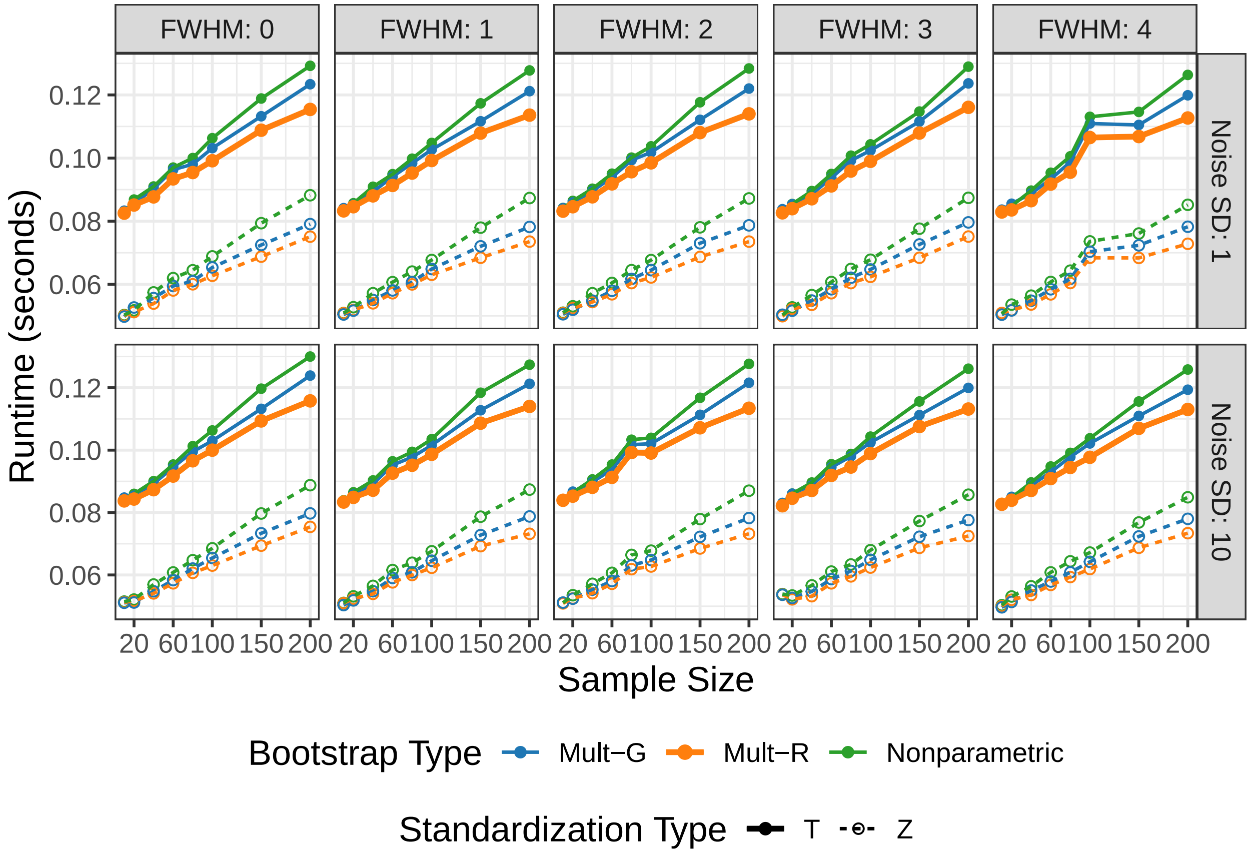

Figure 2.9: Runtime results in scenarios with ellipse shape, Gaussian noise distribution and image size of 50×50. Six bootstrap methods (3 bootstrap types $\times$ 2 standardization types) were evaluated. Confidence intervals of runtime are not plotted since they are extremely narrow, due to the runtime being highly consistent across the 1,000 simulations.

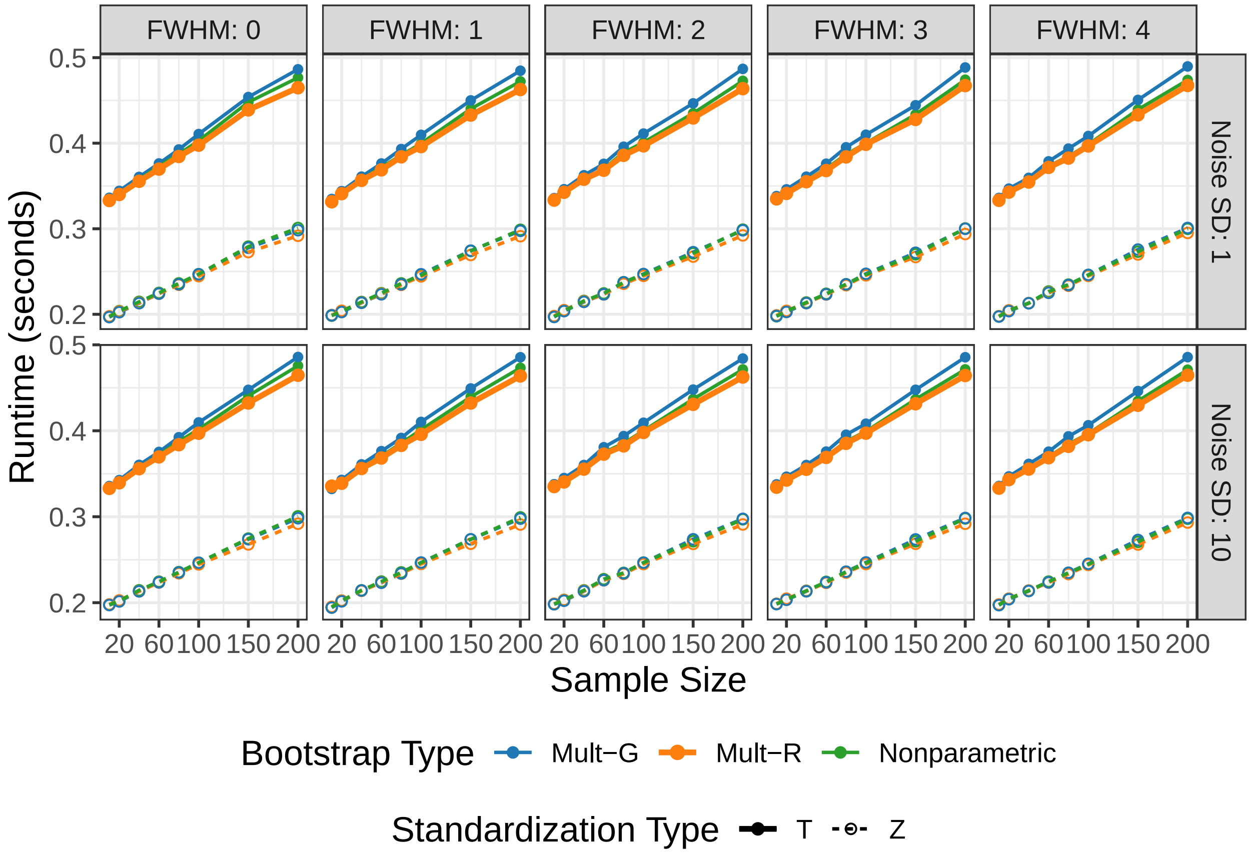

Figure 2.10: Runtime results in scenarios with ellipse shape, Gaussian noise distribution and image size of 100×100. Six bootstrap methods (3 bootstrap types $\times$ 2 standardization types) were evaluated. Confidence intervals of runtime are not plotted since they are extremely narrow, due to the runtime being highly consistent across the 1,000 simulations.

2.3 Precision

The plots below compare precision of SCBs in all simulated scenarios, where a smaller mean quantile represents a more precise SCB. Of note, only methods achieving a relatively good coverage rate were shown here since precision is irrelevant for methods with poor coverage.

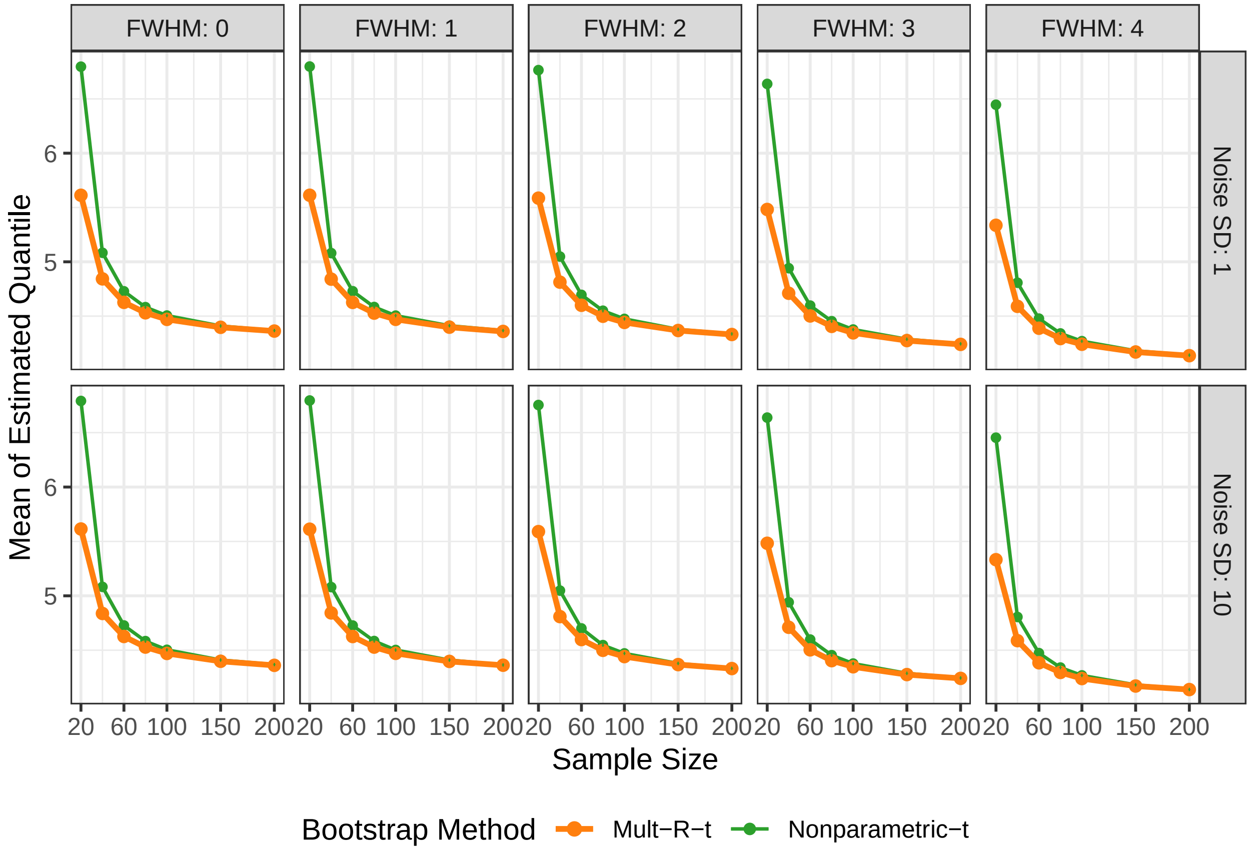

Figure 2.11: Precision results in scenarios with ellipse shape, gaussian noise distribution and image size of 50 $\times$ 50. Two bootstrap methods that achieved a good coverage rate were compared. A smaller mean quantile represents a narrower (i.e., more precise) SCB. The error bars represent 95% confidence intervals (CIs). Some CIs are very narrow and may not be visible on the plot.

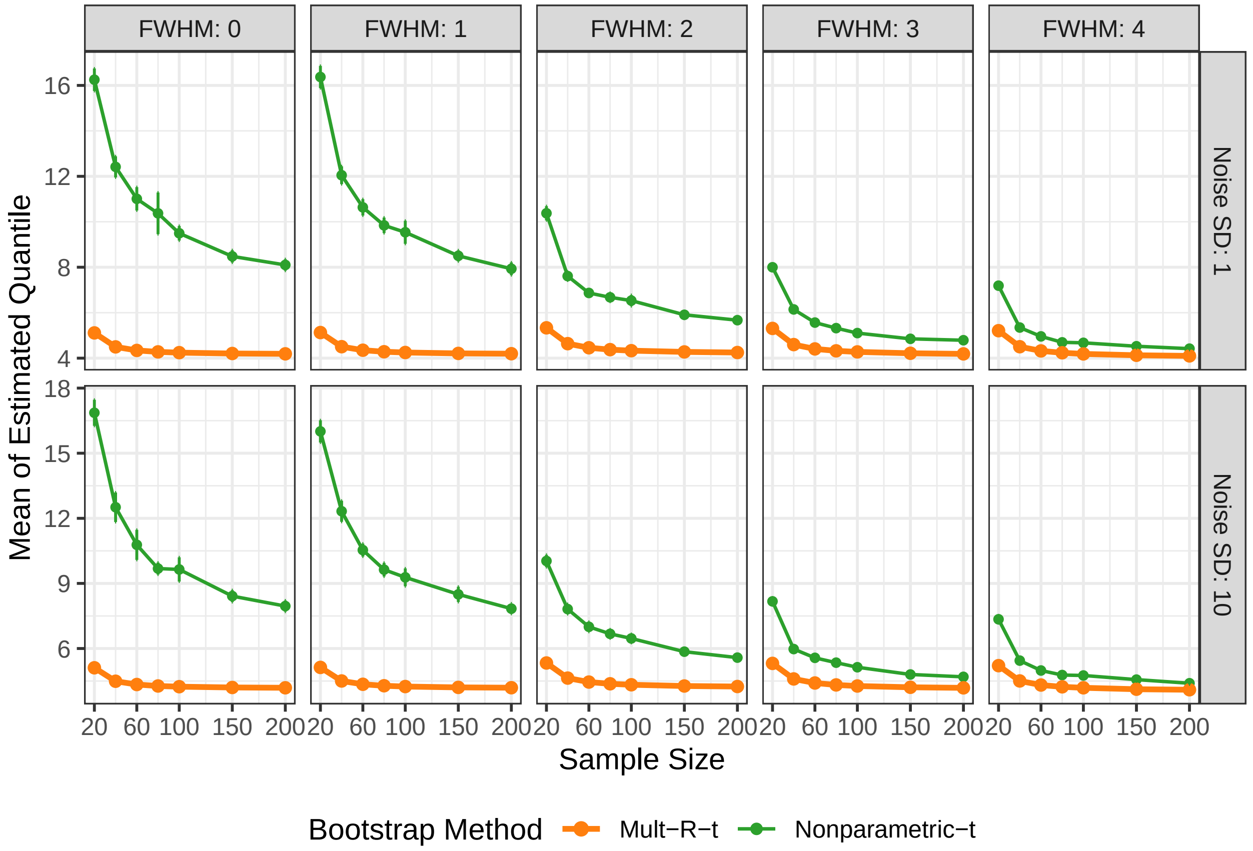

Figure 2.12: Precision results in scenarios with ellipse shape, t noise distribution and image size of 50 × 50. Two bootstrap methods that achieved a good coverage rate were compared. A smaller mean quantile represents a narrower (i.e., more precise) SCB. The error bars represent 95% confidence intervals (CIs). Some CIs are very narrow and may not be visible on the plot.

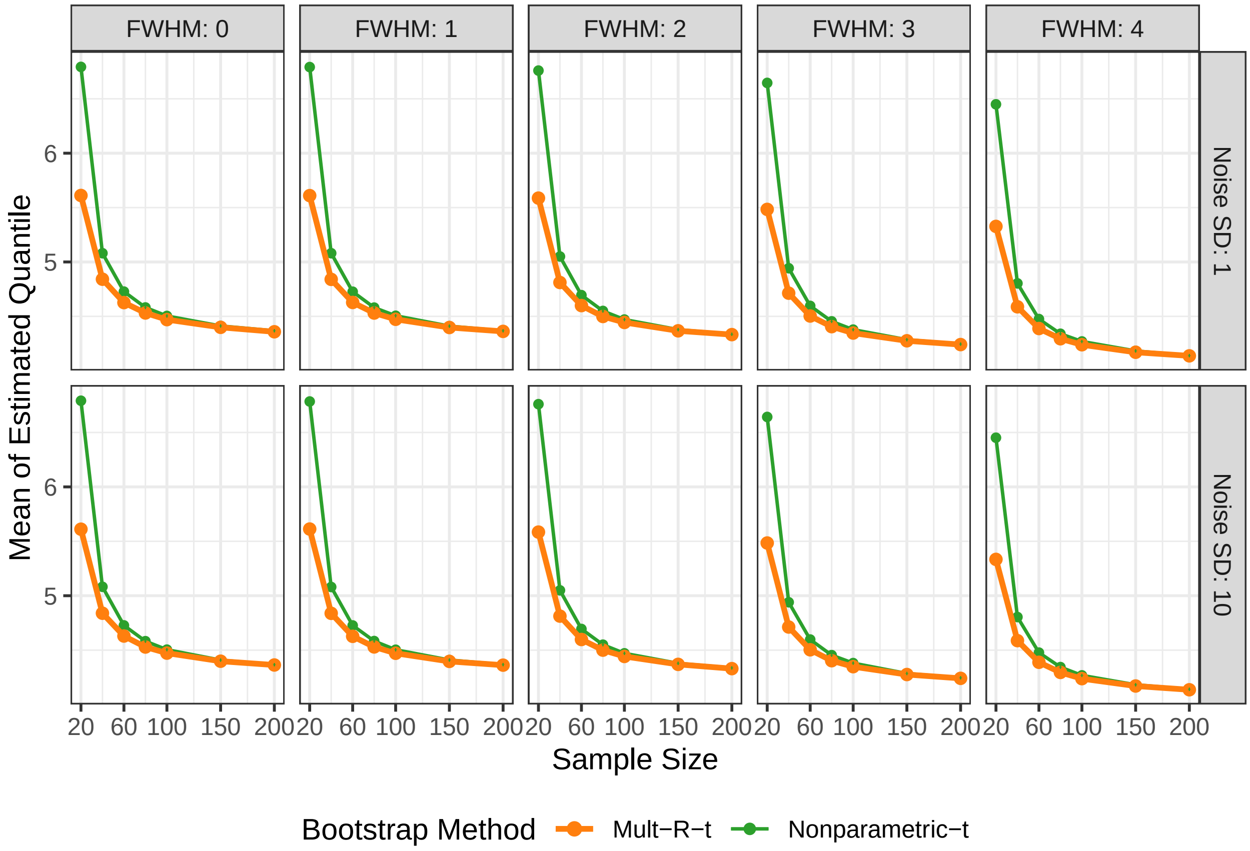

Figure 2.13: Precision results in scenarios with ramp shape, gaussian noise distribution and image size of 50 × 50. Two bootstrap methods that achieved a good coverage rate were compared. A smaller mean quantile represents a narrower (i.e., more precise) SCB. The error bars represent 95% confidence intervals (CIs). Some CIs are very narrow and may not be visible on the plot.

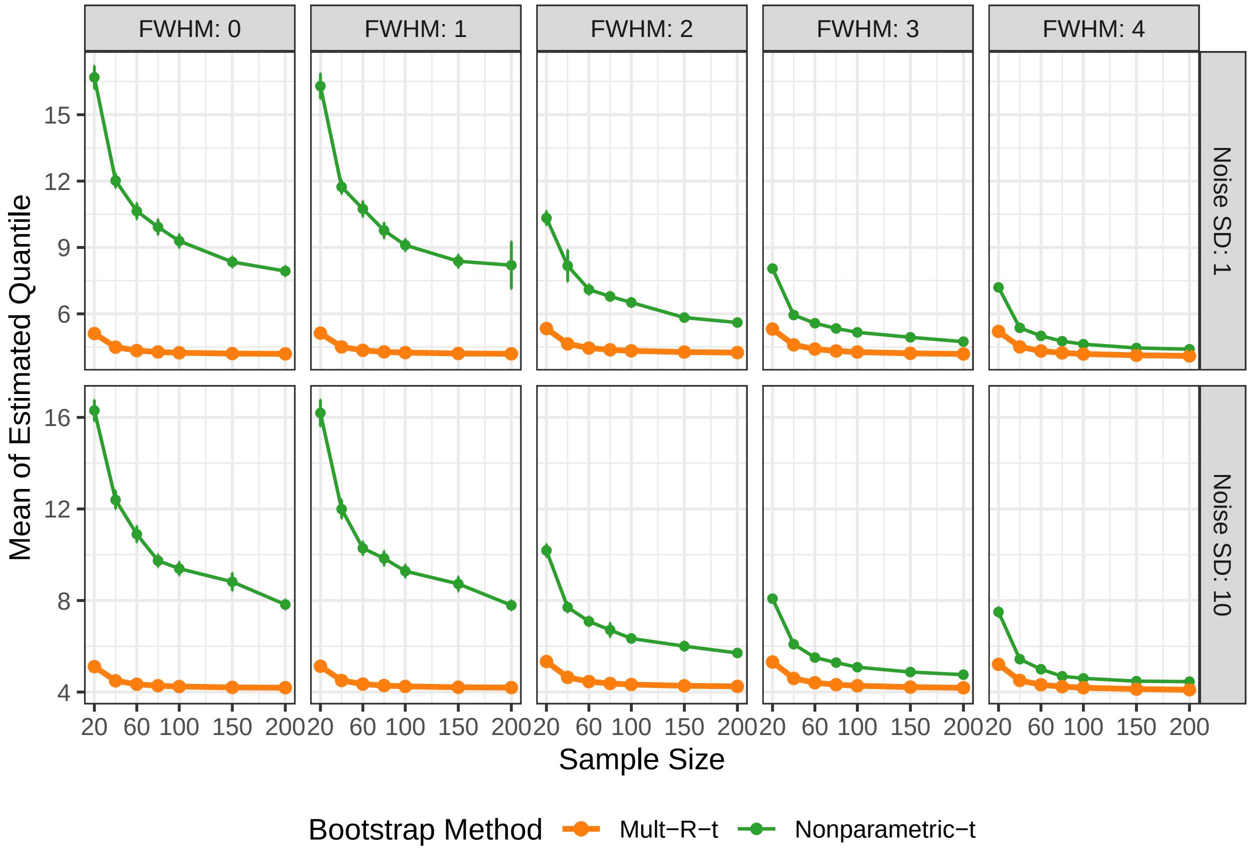

Figure 2.14: Precision results in scenarios with ramp shape, t noise distribution and image size of 50 × 50. Two bootstrap methods that achieved a good coverage rate were compared. A smaller mean quantile represents a narrower (i.e., more precise) SCB. The error bars represent 95% confidence intervals (CIs). Some CIs are very narrow and may not be visible on the plot.

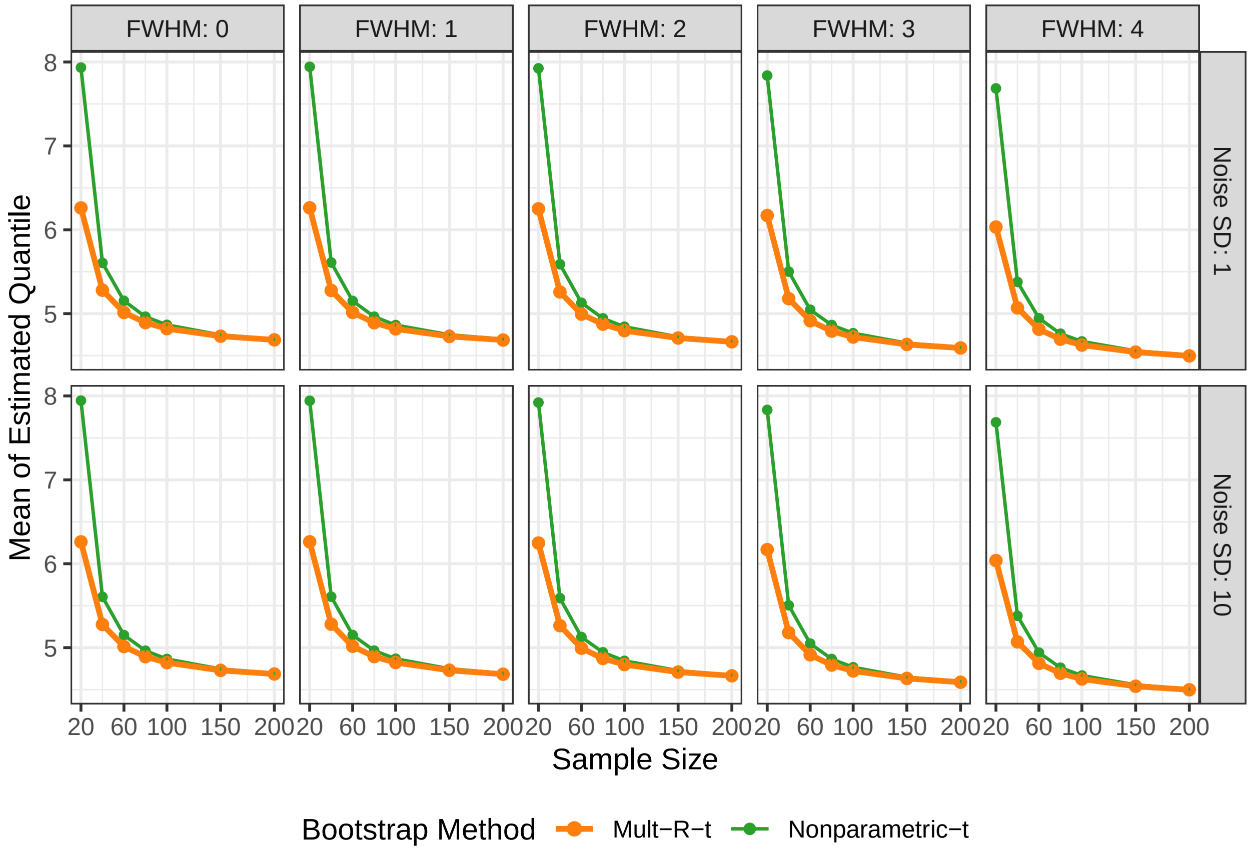

Figure 2.15: Precision results in scenarios with ellipse shape, gaussian noise distribution and image size of 100 × 100. Two bootstrap methods that achieved a good coverage rate were compared. A smaller mean quantile represents a narrower (i.e., more precise) SCB. The error bars represent 95% confidence intervals (CIs). Some CIs are very narrow and may not be visible on the plot.

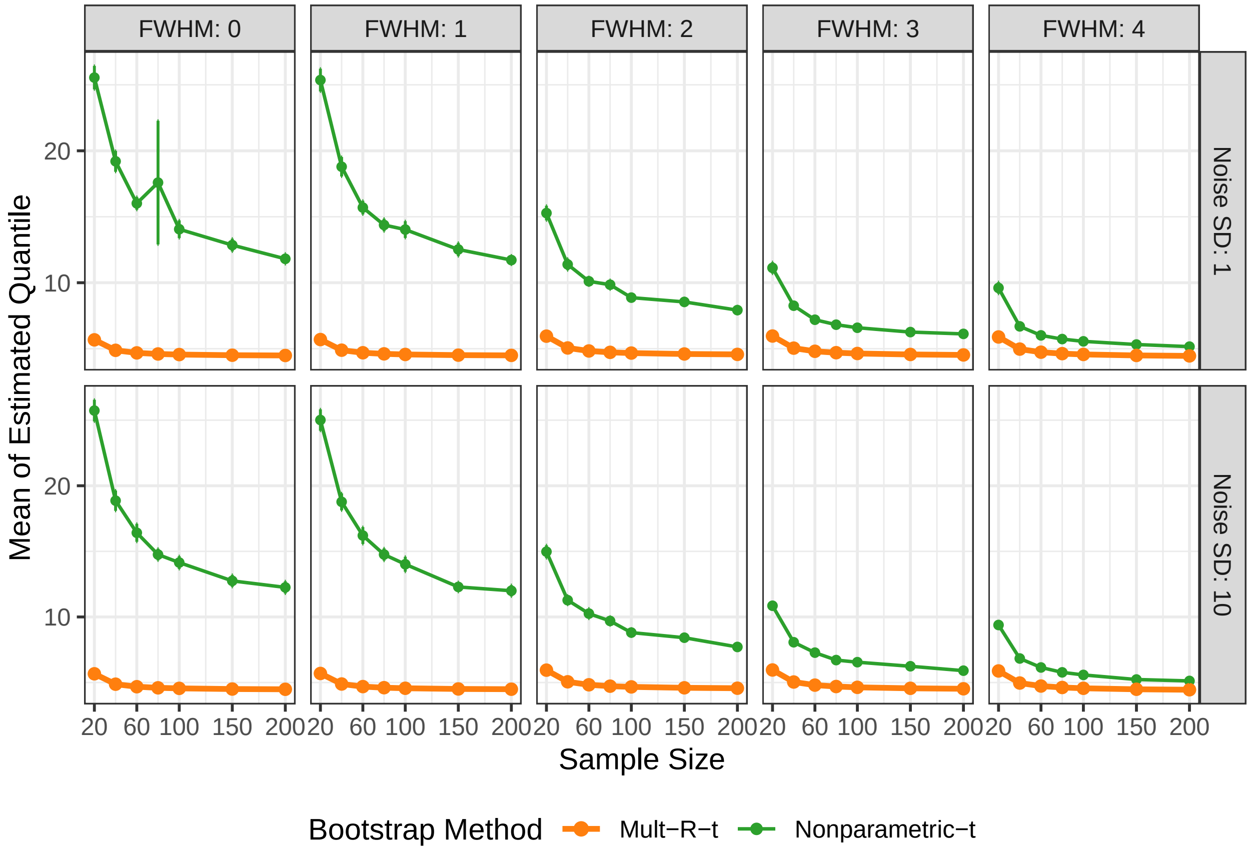

Figure 2.16: Precision results in scenarios with ellipse shape, t noise distribution and image size of 100 × 100. Two bootstrap methods that achieved a good coverage rate were compared. A smaller mean quantile represents a narrower (i.e., more precise) SCB. The error bars represent 95% confidence intervals (CIs). Some CIs are very narrow and may not be visible on the plot.

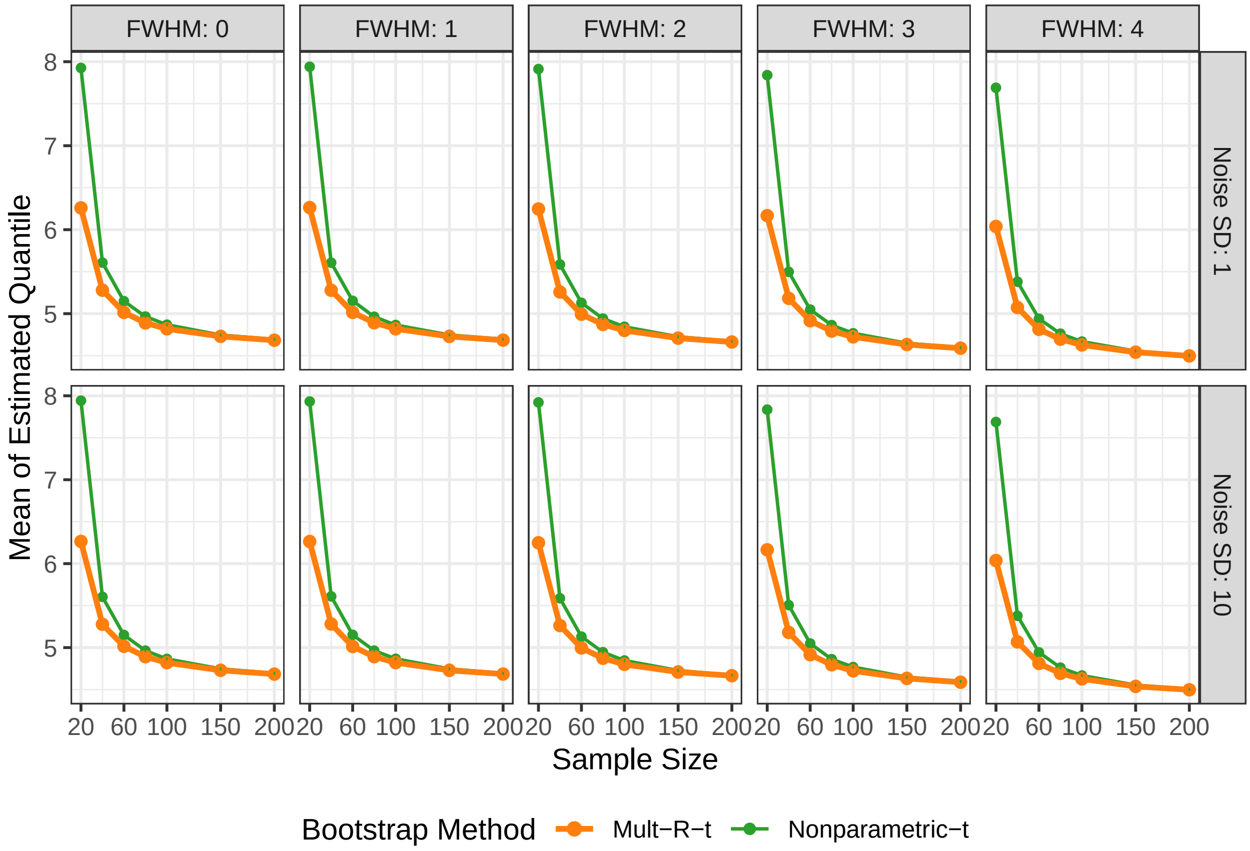

Figure 2.17: Precision results in scenarios with ramp shape, gaussian noise distribution and image size of 100 × 100. Two bootstrap methods that achieved a good coverage rate were compared. A smaller mean quantile represents a narrower (i.e., more precise) SCB. The error bars represent 95% confidence intervals (CIs). Some CIs are very narrow and may not be visible on the plot.

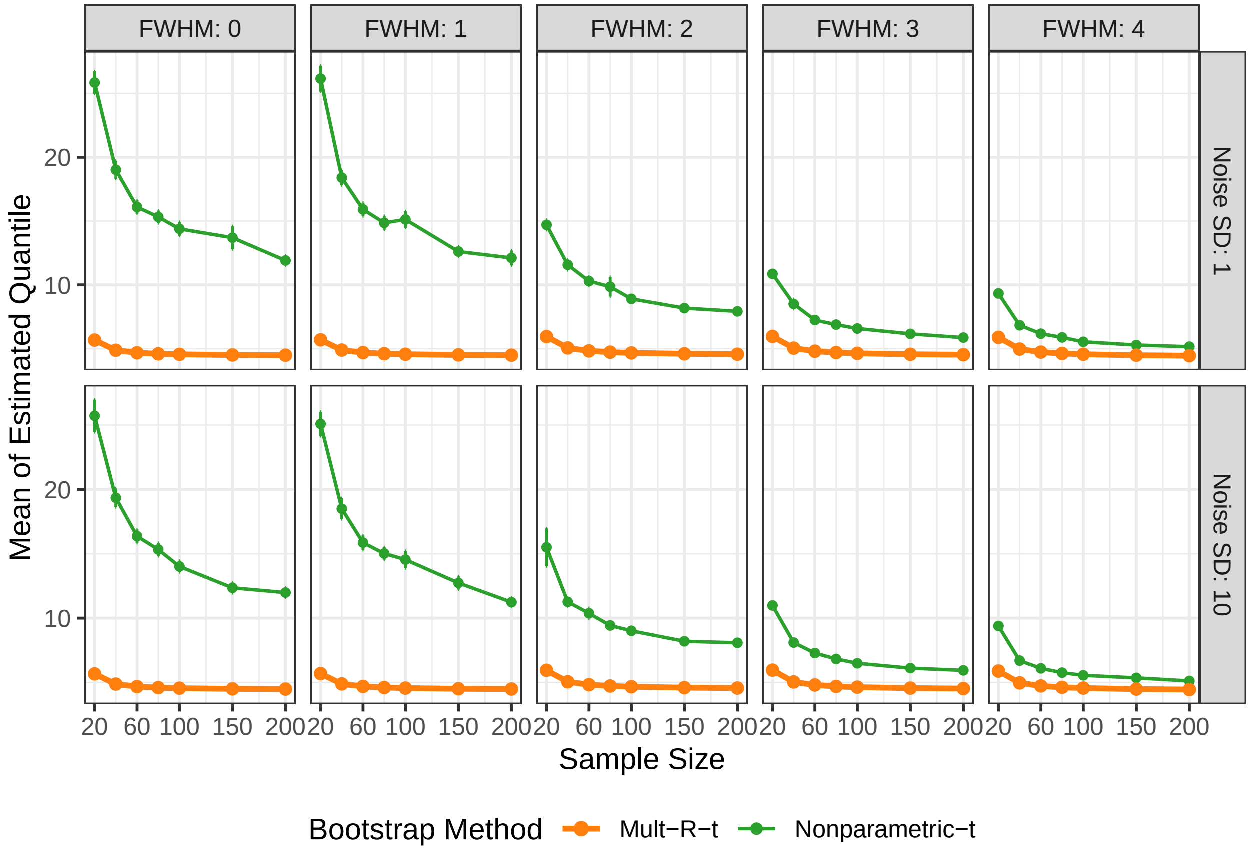

Figure 2.18: Precision results in scenarios with ramp shape, t noise distribution and image size of 100 × 100. Two bootstrap methods that achieved a good coverage rate were compared. A smaller mean quantile represents a narrower (i.e., more precise) SCB. The error bars represent 95% confidence intervals (CIs). Some CIs are very narrow and may not be visible on the plot.

2.4 Stability

The plots below compare stability of SCBs in all simulated scenarios, where a smaller SD of quantile represents a more stable SCB. Of note, only methods achieving a relatively good coverage rate were shown here since stability is irrelevant for methods with poor coverage.

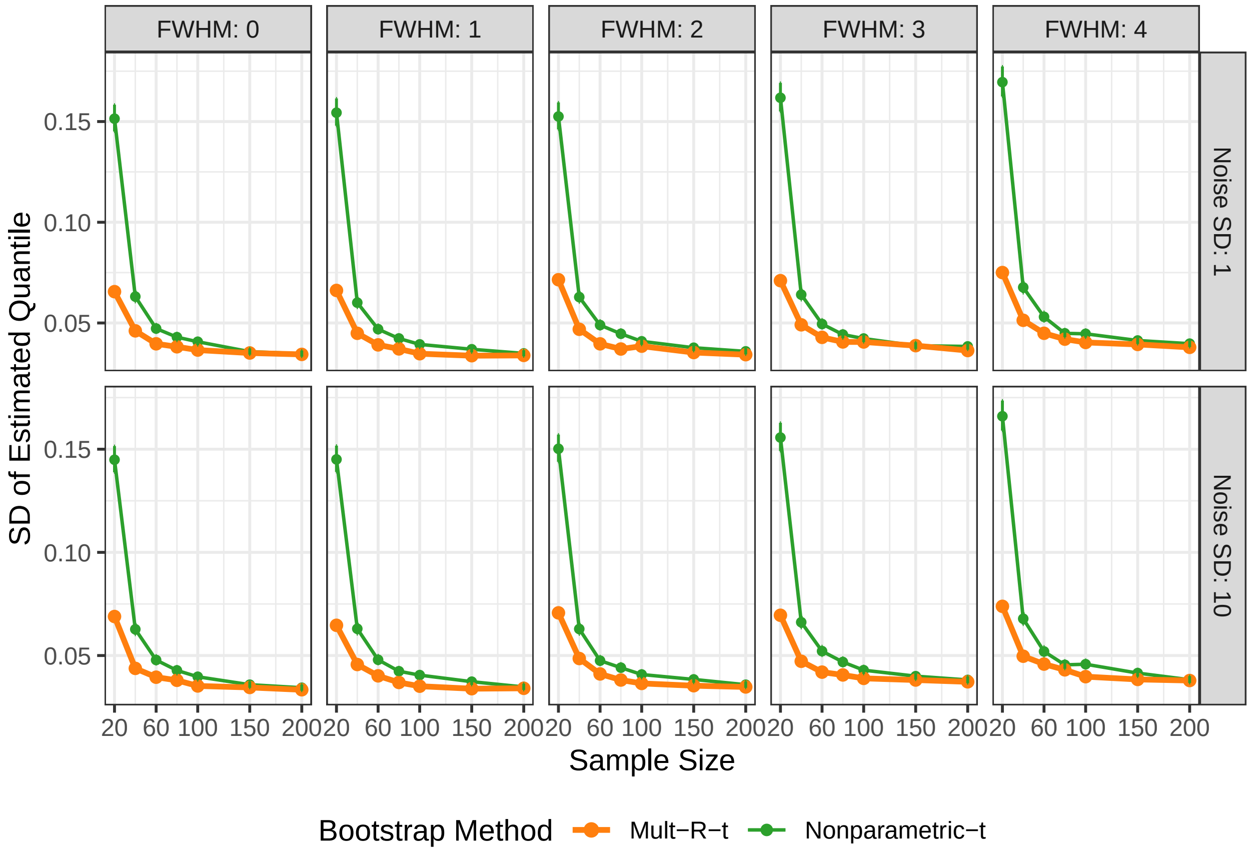

Figure 2.19: Stability results in scenarios with ellipse shape, gaussian noise distribution and image size of 50 × 50. Two bootstrap methods that achieved a good coverage rate were compared. A smaller SD of quantiles represents a more stable SCB. The error bars represent 95% confidence intervals (CIs). Some CIs are extremely narrow and thus may not be visible on the plot.

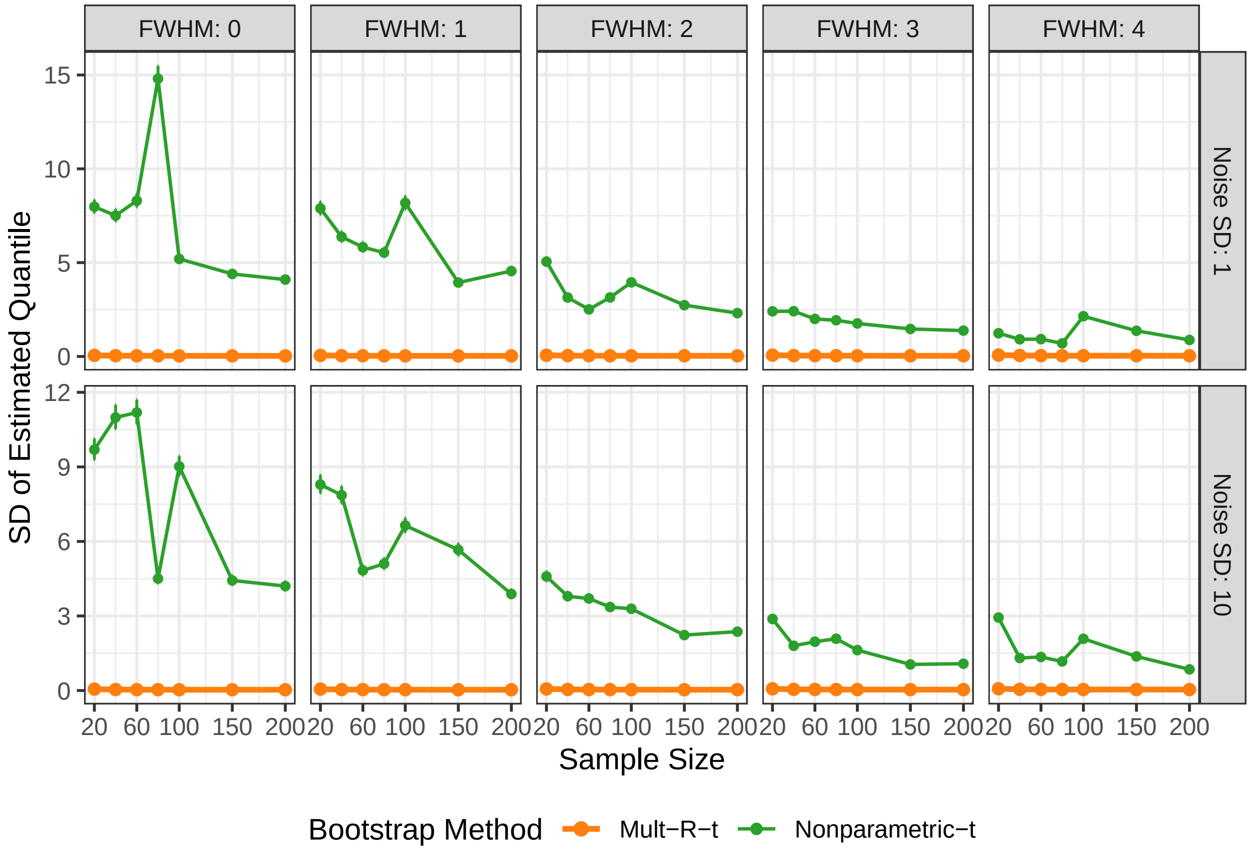

Figure 2.20: Stability results in scenarios with ellipse shape, t noise distribution and image size of 50 × 50. Two bootstrap methods that achieved a good coverage rate were compared. A smaller SD of quantiles represents a more stable SCB. The error bars represent 95% confidence intervals (CIs). Some CIs are extremely narrow and thus may not be visible on the plot.

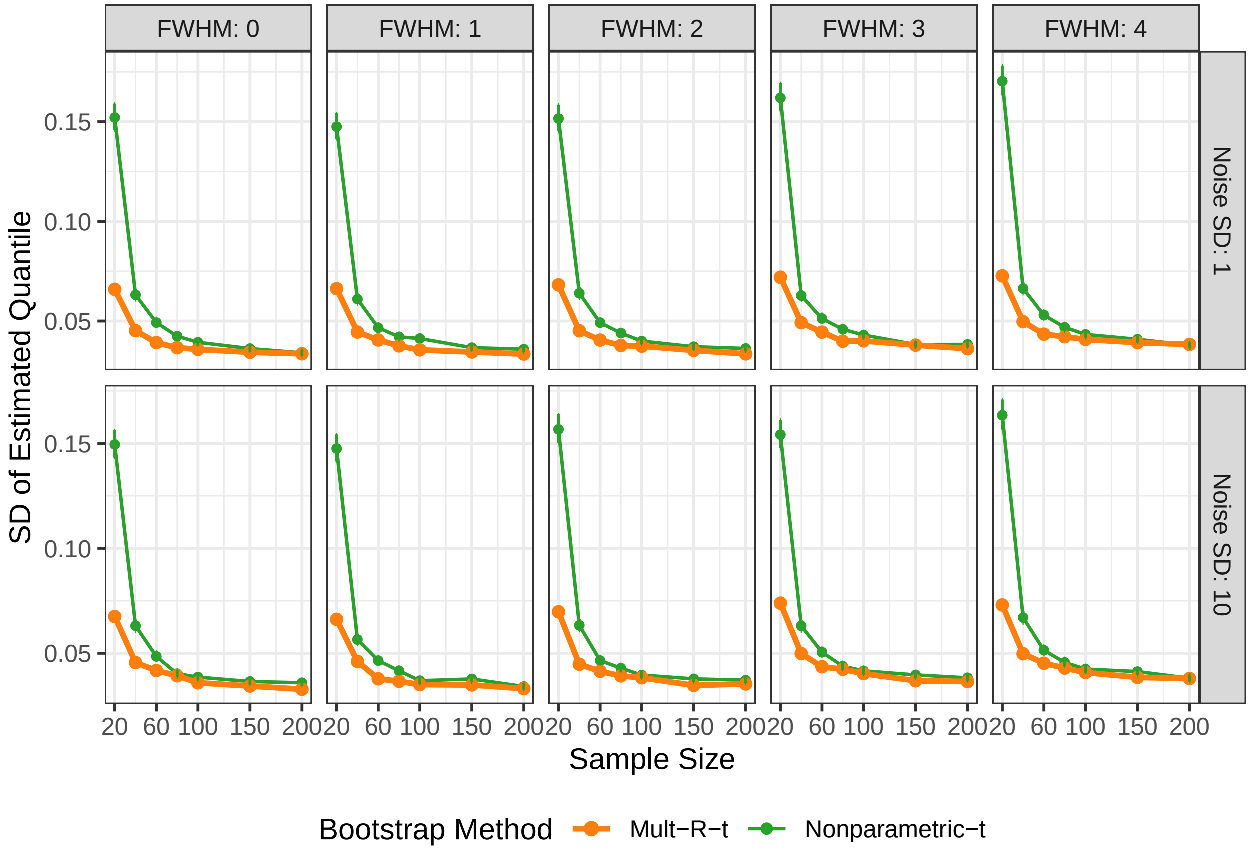

Figure 2.21: Stability results in scenarios with ramp shape, gaussian noise distribution and image size of 50×50. Two bootstrap methods that achieved a good coverage rate were compared. A smaller SD of quantiles represents a more stable SCB. The error bars represent 95% confidence intervals (CIs). Some CIs are extremely narrow and thus may not be visible on the plot.

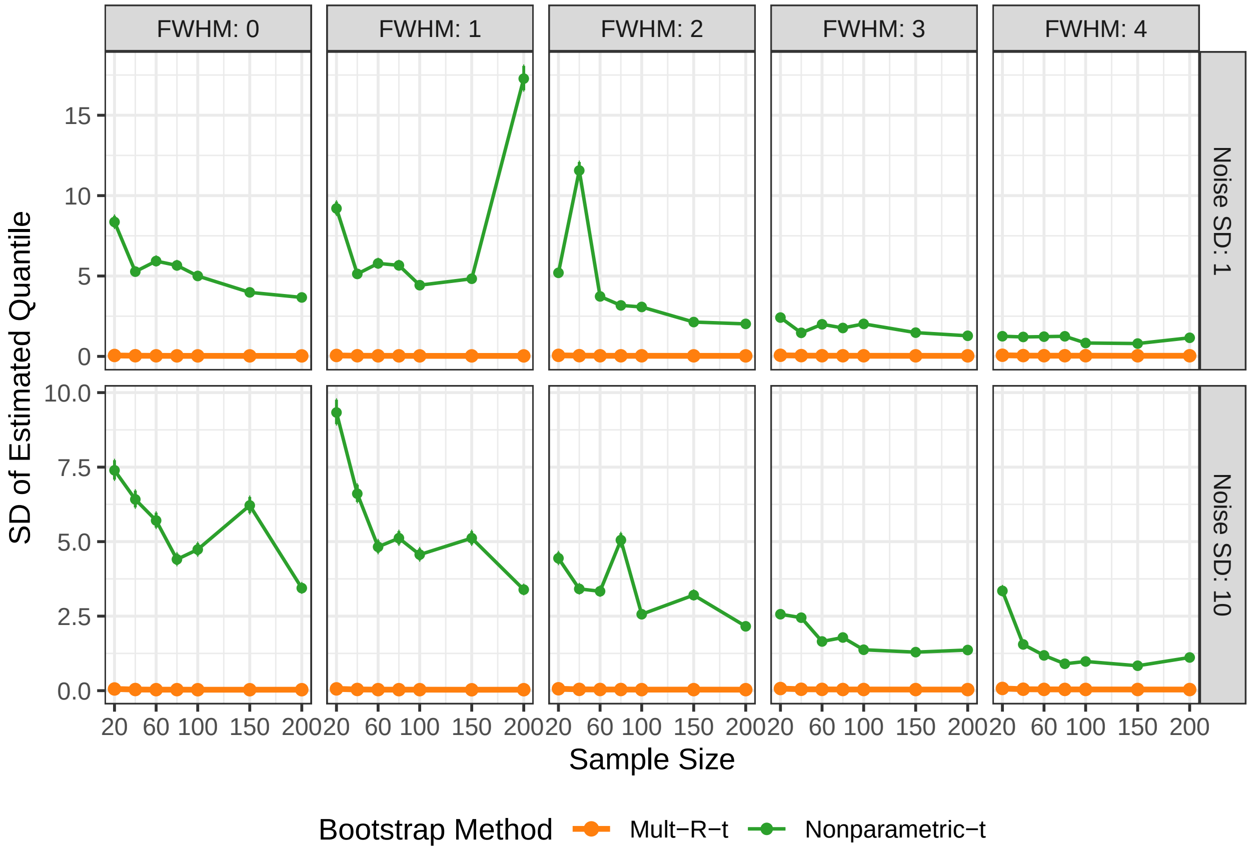

Figure 2.22: Stability results in scenarios with ramp shape, t noise distribution and image size of 50 × 50. Two bootstrap methods that achieved a good coverage rate were compared. A smaller SD of quantiles represents a more stable SCB. The error bars represent 95% confidence intervals (CIs). Some CIs are extremely narrow and thus may not be visible on the plot.

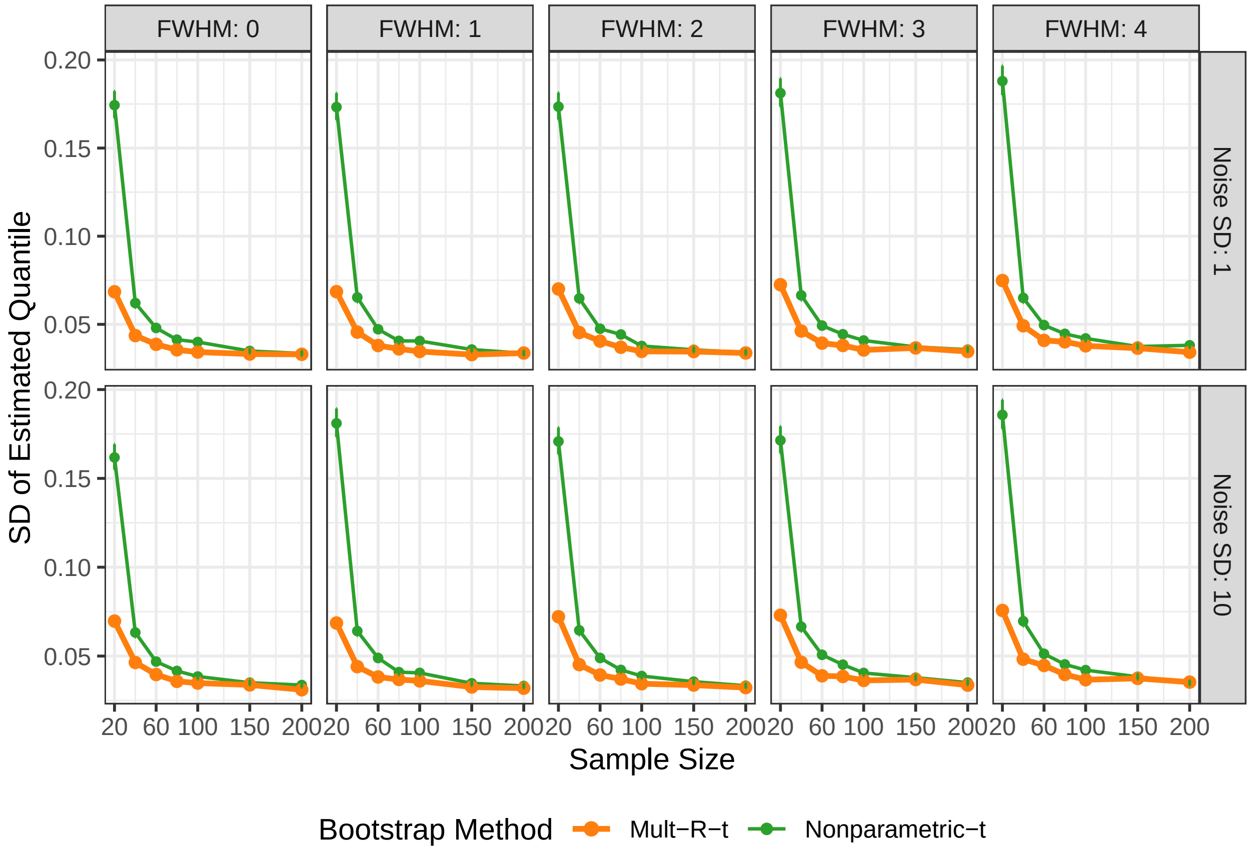

Figure 2.23: Stability results in scenarios with ellipse shape, gaussian noise distribution and image size of 100×100. Two bootstrap methods that achieved a good coverage rate were compared. A smaller SD of quantiles represents a more stable SCB. The error bars represent 95% confidence intervals (CIs). Some CIs are extremely narrow and thus may not be visible on the plot.

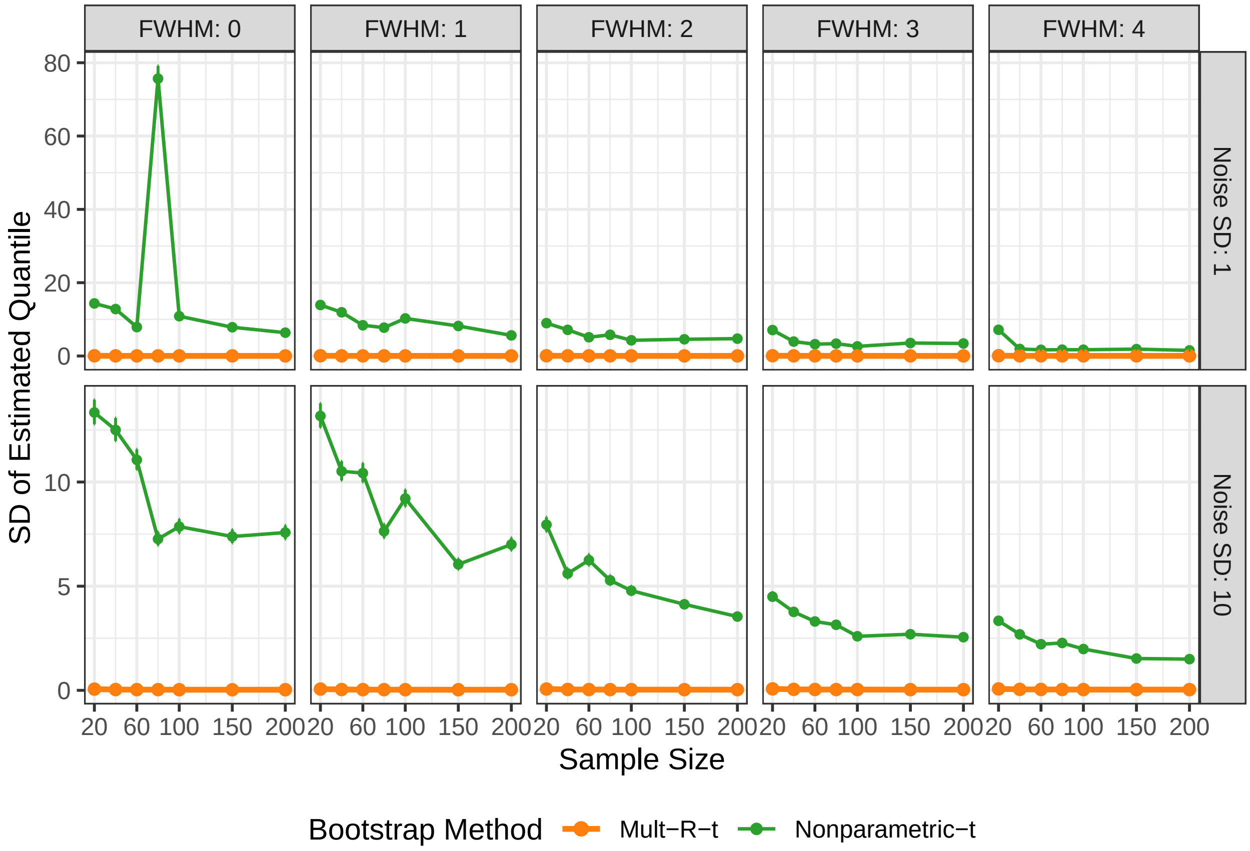

Figure 2.24: Stability results in scenarios with ellipse shape, t noise distribution and image size of 100 × 100. Two bootstrap methods that achieved a good coverage rate were compared. A smaller SD of quantiles represents a more stable SCB. The error bars represent 95% confidence intervals (CIs). Some CIs are extremely narrow and thus may not be visible on the plot.

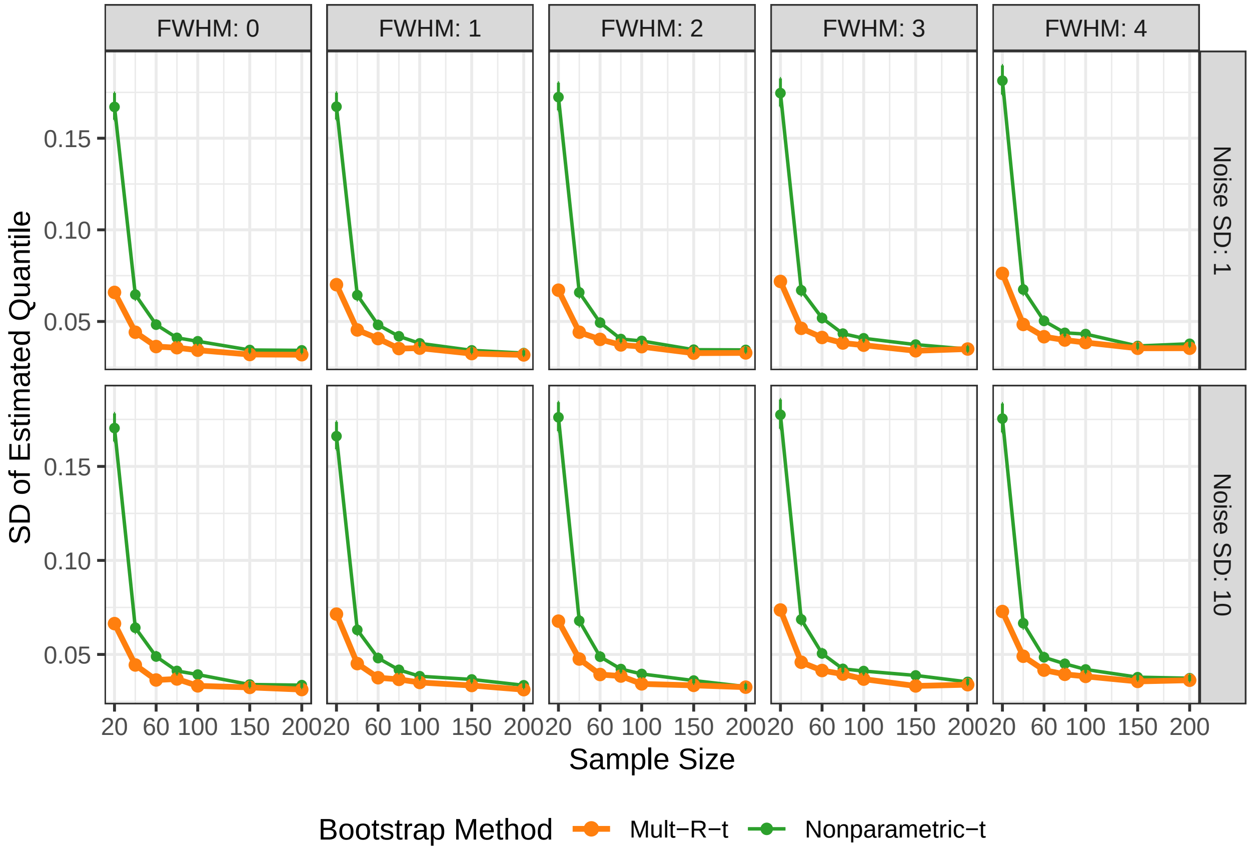

Figure 2.25: Stability results in scenarios with ramp shape, gaussian noise distribution and image size of 100 × 100. Two bootstrap methods that achieved a good coverage rate were compared. A smaller SD of quantiles represents a more stable SCB. The error bars represent 95% confidence intervals (CIs). Some CIs are extremely narrow and thus may not be visible on the plot.

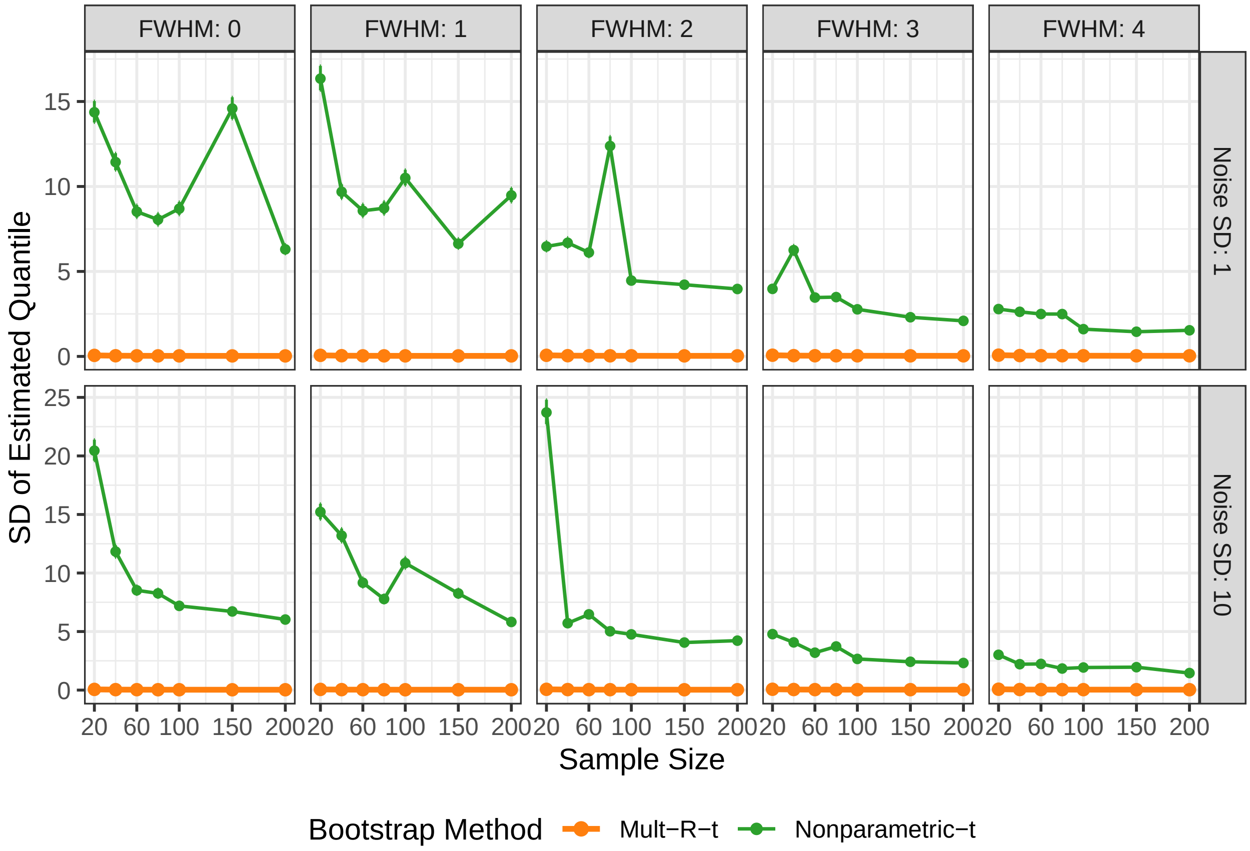

Figure 2.26: Stability results in scenarios with ramp shape, t noise distribution and image size of 100 × 100. Two bootstrap methods that achieved a good coverage rate were compared. A smaller SD of quantiles represents a more stable SCB. The error bars represent 95% confidence intervals (CIs). Some CIs are extremely narrow and thus may not be visible on the plot.

2.5 Impact of Different Numbers of Bootstrap Samples

The plots below are results when different numbers of bootstrap samples were used.

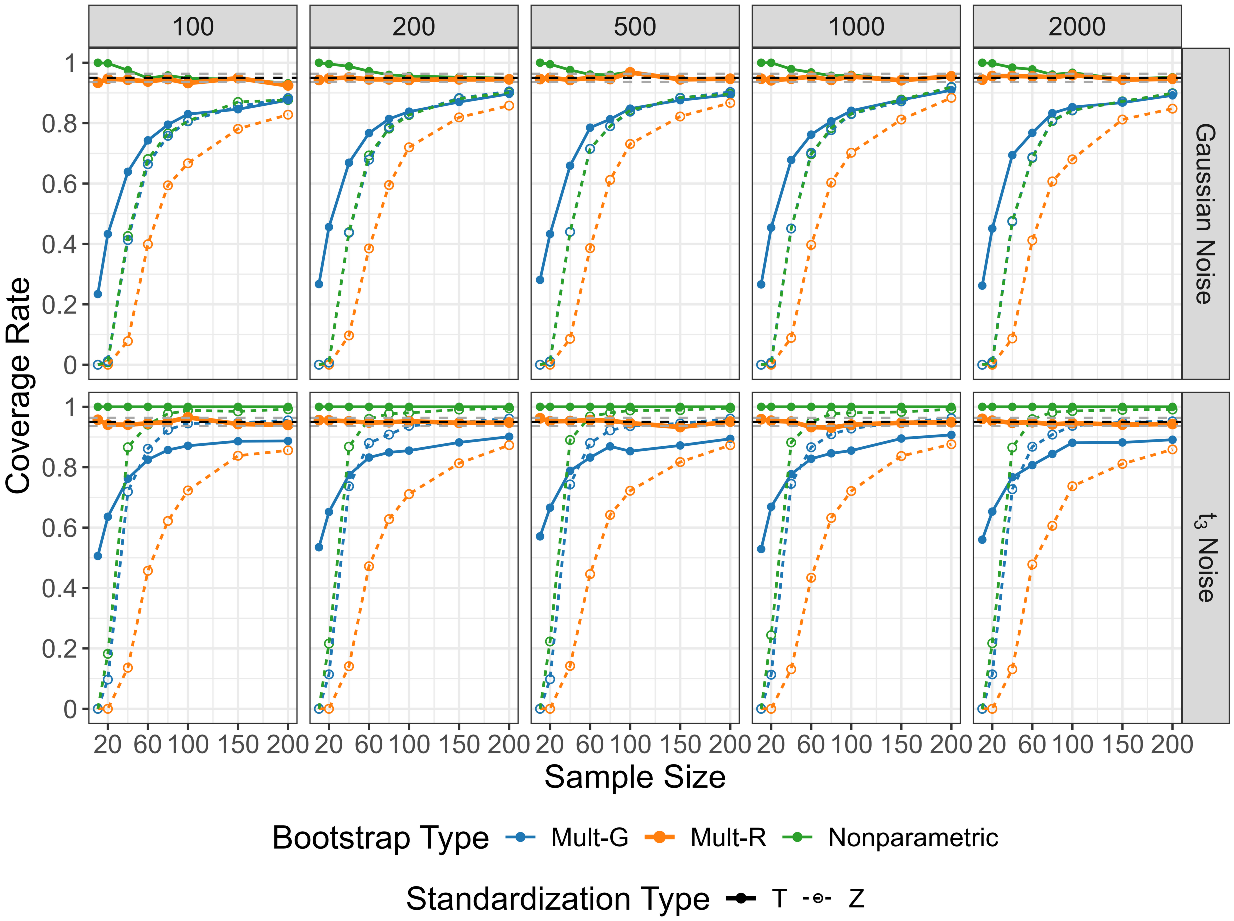

Figure 2.27: Coverage results of 2D simulations under variations in sample size, noise distribution and number of bootstrap samples (100, 200, 500, 1000, 2000). Six bootstrap methods (3 bootstrap types × 2 standardization types) were evaluated. The black dashed line represents the target coverage rate of 0.95. The two gray dashed lines capture the uncertainty due to simulation and correspond to $0.95 \pm 1.96 \times \sqrt{0.95(1-0.95)/1000}$.

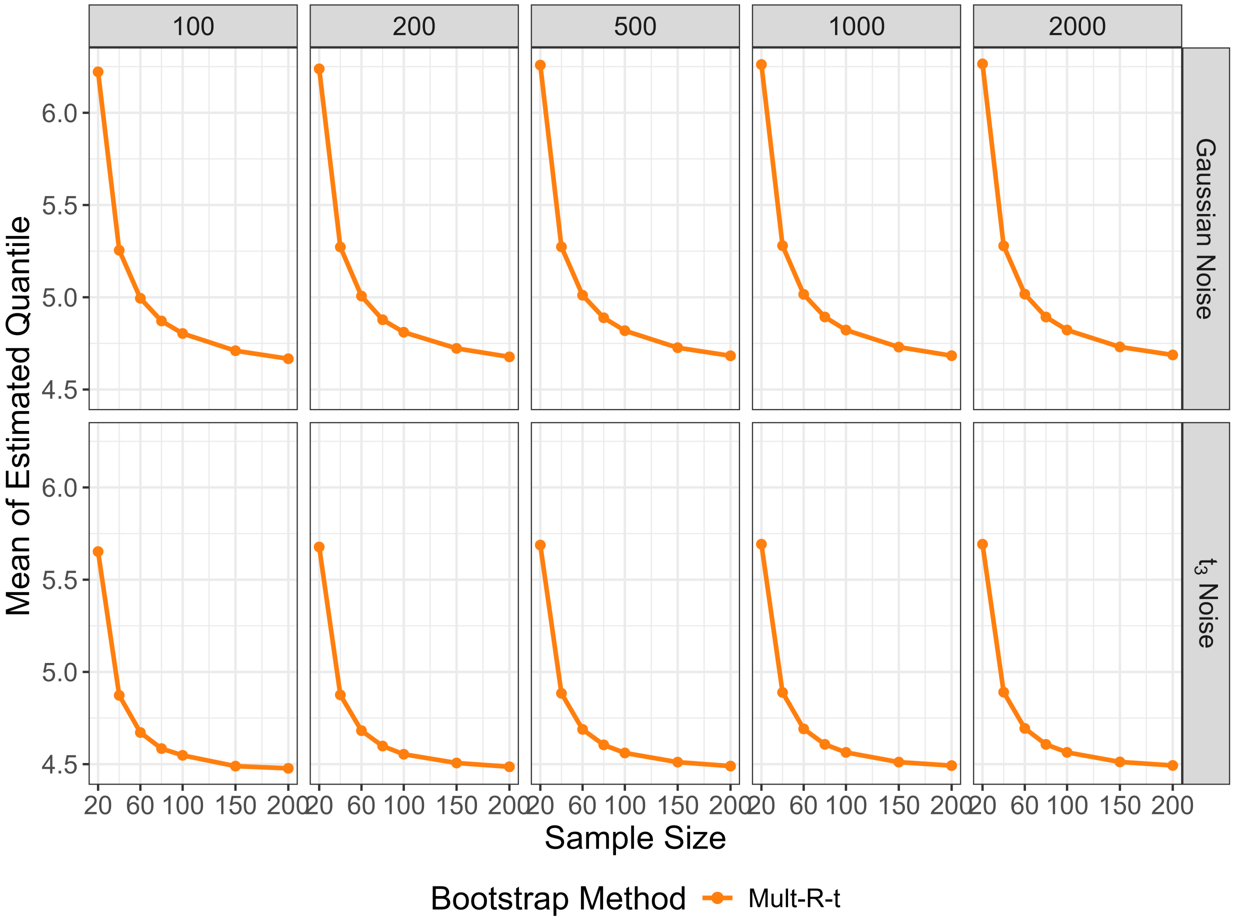

Figure 2.28: Results of 2D simulations on mean of estimated SCB quantiles under variations in sample size, noise distribution and number of bootstrap samples (100, 200, 500, 1000, 2000). SCBs were obtained using Rademacher multiplier bootstrap-t. A smaller mean quantile represents a narrower (i.e., more precise) SCB.

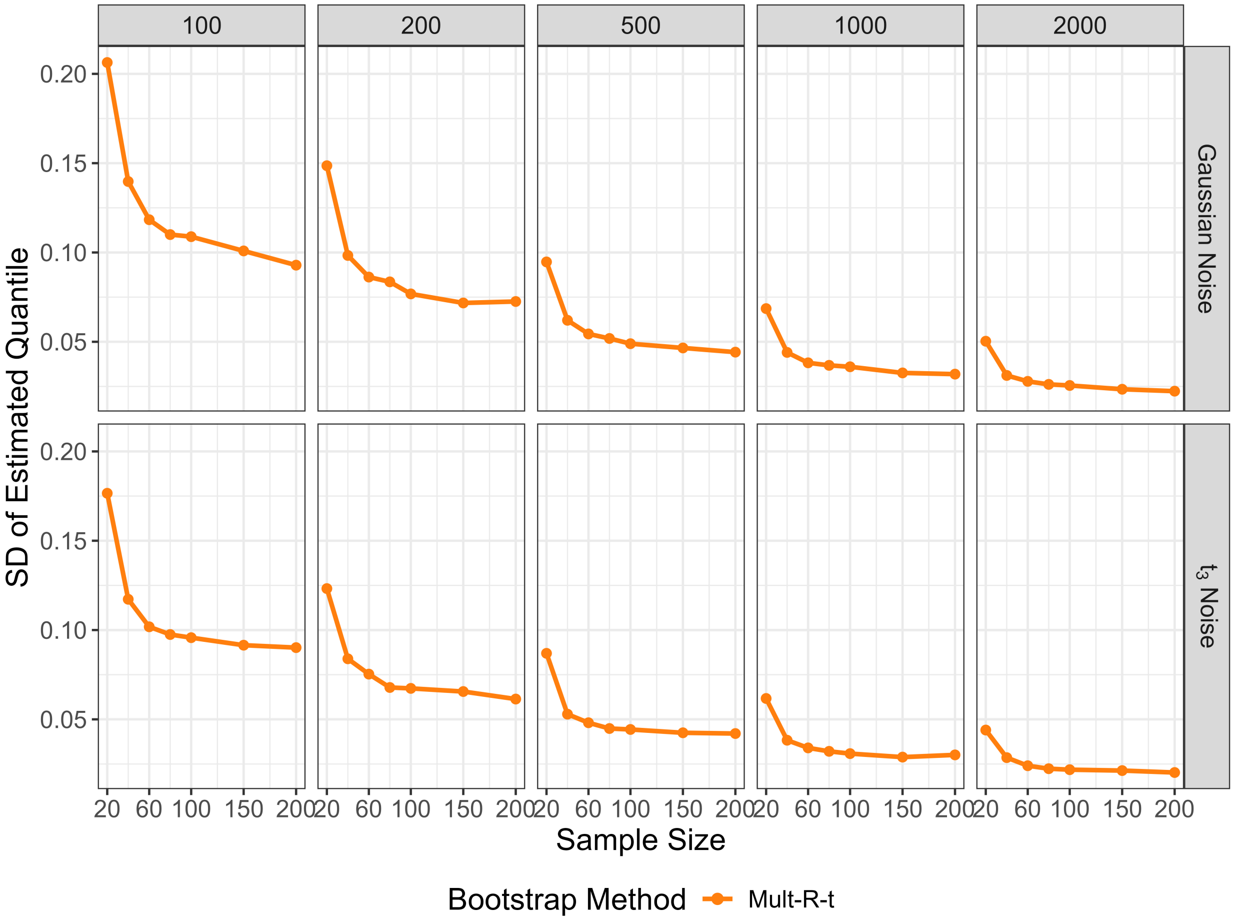

Figure 2.29: Results of 2D simulations on SD of estimated SCB quantiles under variations in sample size, noise distribution and number of bootstrap samples (100, 200, 500, 1000, 2000). SCBs were obtained using Rademacher multiplier bootstrap-t. A smaller SD of quantile represents a more stable SCB.

2.6 Skewed Noise Distributions

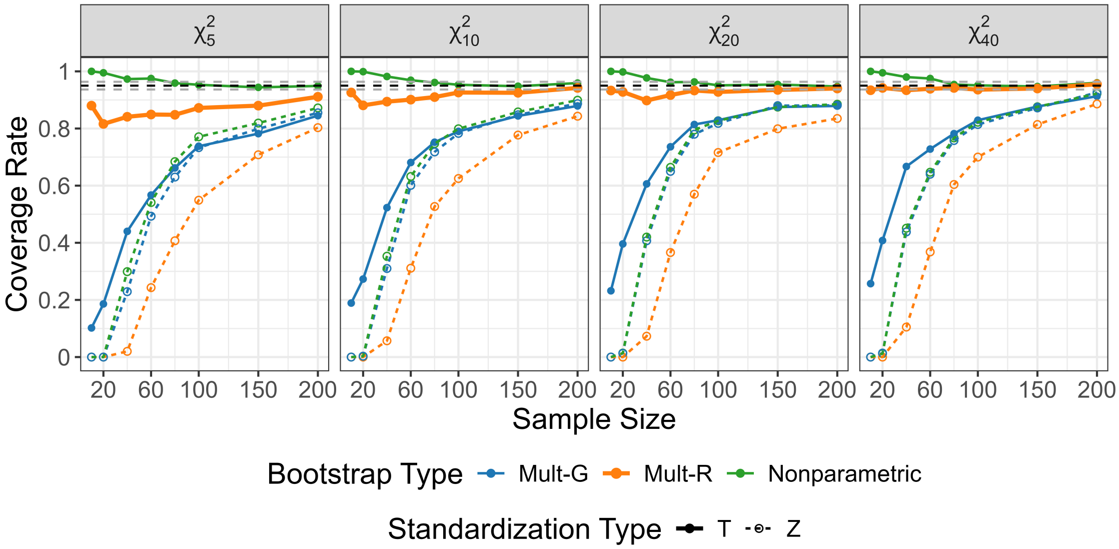

Figure 2.30: Coverage results of 2D simulations under scenarios with a 2D image of size 100 × 100, ellipse-shaped signal, χ^2 noise, FWHM smoothing of 2 and SD of 10. Note that χ^2 distributions with smaller degrees of freedom are more skewed. Six bootstrap methods (3 bootstrap types × 2 standardization types) were evaluated. The black dashed line represents the target coverage rate of 0.95. The two gray dashed lines capture the uncertainty due to simulation and correspond to $0.95 \pm 1.96 \times \sqrt{0.95(1-0.95)/1000}$.
